# Adherence to Mediterranean Diet and Risk of Cancer: An Updated Systematic Review and Meta-Analysis

**DOI:** 10.3390/nu9101063

**Published:** 2017-09-26

**Authors:** Lukas Schwingshackl, Carolina Schwedhelm, Cecilia Galbete, Georg Hoffmann

**Affiliations:** 1 Department of Epidemiology, German Institute of Human Nutrition Potsdam-Rehbruecke (DIfE), Arthur-Scheunert-Allee 114-116, 14558 Nuthetal, Germany; carolina.schwedhelm@dife.de; 2 Department of Molecular Epidemiology, German Institute of Human Nutrition Potsdam-Rehbruecke, 14558 Nuthetal, Germany; Cecilia.Galbete@dife.de; 3 Department of Nutritional Sciences, University of Vienna, Althanstraße 14, 1090 Vienna, Austria; georg.hoffmann@univie.ac.at

**Keywords:** Mediterranean Diet, cancer, meta-analysis, systematic review update

## Abstract

The aim of the present systematic review and meta-analysis was to gain further insight into the effects of adherence to Mediterranean Diet (MedD) on risk of overall cancer mortality, risk of different types of cancer, and cancer mortality and recurrence risk in cancer survivors. Literature search was performed using the electronic databases PubMed, and Scopus until 25 August 2017. We included randomized trials (RCTs), cohort (for specific tumors only incidence cases were used) studies, and case-control studies. Study-specific risk ratios, hazard ratios, and odds ratios (RR/HR/OR) were pooled using a random effects model. Observational studies (cohort and case-control studies), and intervention trials were meta-analyzed separately. The updated review process showed 27 studies that were not included in the previous meta-analysis (total number of studies evaluated: 83 studies). An overall population of 2,130,753 subjects was included in the present update. The highest adherence score to a MedD was inversely associated with a lower risk of cancer mortality (RR_cohort_: 0.86, 95% CI 0.81 to 0.91, *I*^2^ = 82%; *n* = 14 studies), colorectal cancer (RR_observational_: 0.82, 95% CI 0.75 to 0.88, *I*^2^ = 73%; *n* = 11 studies), breast cancer (RR_RCT_: 0.43, 95% CI 0.21 to 0.88, *n* = 1 study) (RR_observational_: 0.92, 95% CI 0.87 to 0.96, *I*^2^ = 22%, *n* = 16 studies), gastric cancer (RR_observational_: 0.72, 95% CI 0.60 to 0.86, *I*^2^ = 55%; *n* = 4 studies), liver cancer (RR_observational_: 0.58, 95% CI 0.46 to 0.73, *I*^2^ = 0%; *n* = 2 studies), head and neck cancer (RR_observational_: 0.49, 95% CI 0.37 to 0.66, *I*^2^ = 87%; *n* = 7 studies), and prostate cancer (RR_observational_: 0.96, 95% CI 0.92 to 1.00, *I*^2^ = 0%; *n* = 6 studies). Among cancer survivors, the association between the adherence to the highest MedD category and risk of cancer mortality, and cancer recurrence was not statistically significant. Pooled analyses of individual components of the MedD revealed that the protective effects appear to be most attributable to fruits, vegetables, and whole grains. The updated meta-analysis confirms an important inverse association between adherence to a MedD and cancer mortality and risk of several cancer types, especially colorectal cancer. These observed beneficial effects are mainly driven by higher intakes of fruits, vegetables, and whole grains. Moreover, we were able to report for the first time a small decrease in breast cancer risk (6%) by pooling seven cohort studies.

## 1. Introduction

Mortality rate due to cancer has been decreasing since the 1990s, most likely due to improved measures of preventive examinations as well as therapeutic interventions. However, this heterogeneous complex of diseases still remains one of the major causes of premature death worldwide as it is ranked second to cardiovascular diseases in current statistics [1]. Cancer incidence is estimated to be 18% by the year 2030 [2].

Tumors are regarded to be an age-dependent phenomenon. Despite this, cancer and other chronic diseases increasingly manifest themselves at a younger age [3]. This emphasizes the fact that the growing incidence of malignant diseases is not exclusively attributable to an increase in life expectancy, but rather due to a number of basic environmental and lifestyle risk factors. Approximately 5–10% of all tumor diseases are caused by genetic predisposition, while the pathogenesis of the remaining 90–95% can be explained by unfavorable environmental conditions or an unhealthy lifestyle [4]. The latter can mainly be characterized by an unbalanced diet, lack of exercise, and consumption of alcohol and tobacco [5].

These are modifiable risk factors, meaning that the manifestation of many types of cancer can be prevented or at least postponed. The World Cancer Research Fund (WCRF) assumes that 3–4 million cases of cancer worldwide might be avoided by adopting a healthier lifestyle [6]. The transition from a detrimental to a beneficial lifestyle cannot always be reduced to a simple formula, e.g., abstention in the case of tobacco consumption. It has been suggested that approximately 30% of cancers can be prevented by a healthy diet [7], however, foods simultaneously contain both ingredients that are protective and others that may cause harm. Some specific bioactive compounds from foods with tumor-preventive potential have been characterized in the past, e.g., polyphenols, *n*-3 fatty acids, or monounsaturated fatty acids [8]. By contrast, alcohol or nitrosamines were identified as ingredients correlated with an increased risk of cancer [9,10,11]. Another very important aspect in this regard is the growing number of cancer survivors. Since its first mention by Fitzhugh Mullan in 1985 [12], this term has not been given a distinct definition. However, it is generally acknowledged to include patients with diagnosed cancer who have carried out all required therapeutic measures and are currently showing no symptoms of disease (in remission). This group of the population still has to cope with side-effects of their specific therapy as well as the threat of recurrence. Due to heightened awareness of the necessity of check-ups and routine examinations as well as evolved diagnostic and therapeutic tools, it is reasonable to speculate that the number of cancer survivors will increase substantially in the years ahead [13,14,15].

Dietary interventions will represent an effective measure in secondary/tertiary prevention or as a part of an adjunctive therapy. Despite this, there are at present very few evidence-based nutritional recommendations for cancer survivors [6,16].

Rather than focusing on single nutrients, the assessment of dietary patterns might be a more adequate approach to clarify the connections between nutrition and cancer. In contrast to associations based on single-nutrient analyses, dietary patterns allow for a simultaneous assessment of favorable and unfavorable components in food as well as their interdependencies [17,18]. The Mediterranean diet (MedD) belongs to the a priori defined dietary patterns. For this type of pattern, adherence to a regimen settled in advance has been extensively assessed and consecutively associated with health parameters [19]. The term MedD was coined by Keys and co-workers after observing significantly lower rates of coronary heart disease in countries bordering the Mediterranean Sea (Cyprus, Greece, and Italy) as compared to The Netherlands, USA, or Finland [20,21].

A MedD is characterized by a high consumption of plant-based foods, especially whole grain products, vegetables, fruits, nuts, and legumes with regular intake of fish and seafood. Eggs, red and processed meat as well as high-fat dairy products are consumed in low amounts [19,22,23]. Additional indicators often used to describe a MedD are moderate alcohol consumption, preferably red wine taken with meals. Fat is predominately provided in the form of olive oil [24,25,26]. Taken together, this results in a dietary pattern with a low content of saturated fatty acids (7–8% of daily total energy consumption) and a total percentage of fat of 30–40% per day [27]. 

Despite the limitations regarding the exact recording of the adherence to a MedD and the small number of studies investigating corresponding pathogenetic mechanisms, systematic reviews and meta-analyses of observational studies provide evidence that a MedD exerts protective functions with respect to tumor incidence and mortality [28,29,30,31]. In a clinical trial, choice of diet orientated towards a Mediterranean pattern was found to be associated with reduced all-cause mortality as well as a 61%-decrease in cancer incidence [32]. In addition, a primary-preventive effect of a MedD supplemented with extra-virgin olive oil on breast cancer was reported [33]. 

We decided to update our previous meta-analyses on the topic of MedD and cancer [28] for the second time due to the following reasons: a number of additional epidemiological studies and randomized controlled trials (RCTs) investigating the correlations between MedD and risk of cancer have meanwhile been published and will therefore allow for a more comprehensive synthesis of the corresponding data; the same applies for studies investigating the effects of MedD in cancer survivors (where the previous analysis did not yield any significant correlation between adherence to MedD and cancer mortality or recurrence); new types of cancer localizations have been taken into consideration. In addition, to gain further insights into the complex relationship between diet and cancer, we analyzed the effects of specific food groups typical of the MedD on tumor incidences in this update.

## 2. Methods

The review protocol of the previous versions of the systematic review is registered in PROSPERO International Prospective Register of Systematic Reviews (https://www.crd.york.ac.uk/prospero/display_record.asp?ID=CRD42013004382).

### 2.1. Data Sources and Searches

A literature search was performed using the electronic databases PubMed (25 August 2017), and Scopus (25 August 2017) using the following search terms for PubMed (Appendix A).

The search strategy had no language restrictions. Moreover, reference lists from reviews, meta-analyses, and the retrieved articles were searched to identify further relevant studies. Literature search was conducted by one author (LS), with questions or uncertainties resolved by discussion with another author (GH).

### 2.2. Study Selection

Cohort studies and case-control studies investigating the association between the MedD and risk of cancer mortality and cancer types in the general population; cancer mortality and cancer recurrence among cancer survivors were included in this update [29].

### 2.3. Data Extraction

The following data were extracted from each study as reported in the previous version [29]: name of first author, country, study name, study design, outcome, population size, follow-up, age at entry, sex, components of score, score range, adjustment factors, and risk estimates (most adjusted Hazard ratio (HR), Risk ratio (RR) or Odds ratio (OR) comparing highest vs. lowest adherence category) with their corresponding 95% CIs.

### 2.4. Definition: Adherence to Mediterranean Diet

The two intervention trials differed regarding food components in the MedD intervention groups. Whereas higher intakes of plant based foods such as vegetables, fruits, nuts and legumes were recommended in the PREDIMED as well as the Lyon Diet Heart study, the recommended fat source in the PREDIMED study was extra virgin olive oil and nuts, whereas in the intervention group in the Lyon Heart Study was supplemented with rapeseed oil margarine [34,35]. 

For observational studies meta-analysis, the lowest adherence to MedD category was compared with the highest MedD category (according to the MedD scores by Trichopoulou [36], Fung [37], or Panagiotakos [38]; with the exception of six studies that used factor analysis or principal component analysis to define the MedD (authors named these MedD pattern): Menotti et al. (2011) [39] (HR: per 1 standard deviation increase), Murtaugh et al. (2008) [40] (OR: fourth vs. first quartile), Cottet et al. (2005) [41] (OR: third vs. first tertile), Bessaoud et al. (2012) [42] (OR per increment of one standard error), Castello et al. (2017a) [43] (OR: fourth vs. first quartile), and Castello et al. (2017b) [44] (OR: fourth vs. first quartile). The maximum ranges of the different MedD scores are reported in Table 1.

### 2.5. Statistical Analysis

The meta-analysis was performed by combining the multivariable adjusted RRs, HR or ORs of the highest compared with the lowest MedD adherence category based on random effects model using the DerSimonian-Laird method, which incorporated both within and between study variability [45]. Because outcomes were not very rare and heterogeneity modeling was deemed important, the random effects model was used.

A second meta-analysis was performed to compare the effects of the different dietary components of the Mediterranean dietary pattern on overall cancer risk, based on data given within the papers included in the present meta-analysis. To evaluate the weighting of each study, the standard error for the logarithm HR/RR/OR of each study was calculated and regarded as the estimated variance of the logarithm HR/RR/OR, using an inverse variance method [45]. For the high vs. low adherence to MedD adherence category studies were grouped according to the different clinical outcomes (overall risk of cancer mortality, and risk of colorectal cancer/breast cancer/prostate cancer/gastric cancer/head and neck cancer/esophageal cancer/pancreatic cancer/liver cancer/ovarian cancer/endometrial cancer/respiratory cancer/bladder cancer/gallbladder cancer/biliary tract cancer/and lymphoma). Observational studies (cohort and case-control studies), and intervention trials were meta-analyzed separately. Meta-analysis was stratified for observational studies by study design: cohort studies, and case-control studies.

Heterogeneity was estimated by the Cochrane Q test together with the *I*^2^ statistic. An *I*^2^ value >50% indicates substantial heterogeneity across studies [46,47]. Potential small-study effects, such as publication bias, were explored using Egger’s test and funnel plots when at least 10 studies were available, as recommended by the Cochrane Handbook [48]. All analyses were conducted using the Review Manager by the Cochrane Collaboration (version 5.3 *Copenhagen: The Nordic Cochrane Centre*) and Stata 12.0 (Stata-Corp, College Station, TX, USA).

## 3. Results

### 3.1. Literature Search and Study Characteristics

The detailed steps of the updated meta-analysis article search (Supplementary Materials Figure S1) and selection process are given as an adapted Preferred Reporting Items for Systematic Reviews and Meta-Analyses (PRISMA) flow diagram [49].

Taken together, 83 studies [32,33,37,39,40,42,43,44,50,51,52,53,54,55,56,57,58,59,60,61,62,63,64,65,66,67,68,69,70,71,72,73,74,75,76,77,78,79,80,81,82,83,84,85,86,87,88,89,90,91,92,93,94,95,96,97,98,99,100,101,102,103,104,105,106,107,108,109,110,111,112,113,114,115,116,117,118,119,120,121,122,123,124] were included in the present systematic review update (27 additional studies (two randomized trials [32,33], 16 cohort studies [50,51,52,53,54,55,56,57,58,59,60,61,62,63,64,65], and nine case-control studies [43,44,66,67,68,69,70,71,72]) were identified that were not included in the previous meta-analyses [28,29]). 

General characteristics of these additional studies are summarized in Table 1. For the present updated meta-analyses, data of new studies were synthesized together with the data of the previous reports. Overall, two RCTs, including 4887 subjects; 51 cohort studies including 2,025,303 subjects (incidence cases; biliary tract: 163; bladder: 1804; breast: 18,782; colorectal: 15,108; endometrial: 1392; esophageal: 848; gallbladder: 77; gastric: 1382; head and neck: 1868; liver: 509; pancreatic: 865; prostate: 29,806; ovarian: 696; respiratory: 9875); and 30 case-control studies with 100,563 subjects met the objectives and were included in the updated meta-analysis. An overall population of 2,130,753 subjects was included in the present update.

### 3.2. Main Outcomes

Documentations of the different clinical outcomes are distributed as follows: overall risk of cancer mortality was evaluated in 14 cohorts and one RCT; breast cancer risk in one RCT, seven cohorts and nine case-control studies; colorectal cancer risk in six cohorts and five case control studies; prostate cancer risk in three cohorts and three case-control studies; gastric cancer risk in two cohorts and two case-control study; head and neck cancer in one RCT, in one cohort study and six case-control studies; endometrial cancer in one cohort and two case-control studies; respiratory cancer in one RCT, and three cohort studies; bladder cancer in two cohort studies; liver cancer, pancreatic cancer and esophageal cancer in one cohort study and one case-control study; ovarian, gallbladder, and biliary tract cancer in one cohort study each; lymphoma in one case control study; cancer mortality among cancer survivors in four cohort studies; cancer recurrence among cancer survivors in one cohort study; and cancer-specific mortality in one cohort study.

Using a random effects model, we found that the highest adherence score to the MedD was inversely associated with a lower risk of overall cancer mortality (RR_cohort_: 0.86, 95% confidence interval (CI) 0.81 to 0.91; *I*^2^ = 81%; *n* = 15 studies) but not among one RCT (RR_RCT_: 0.75, 95% CI 0.17 to 3.33, *I*^2^ = NA) (Supplementary Materials Figure S2, Table S2). Among cancer survivors, we observed no association between the adherence to the highest MedD category and risk of cancer mortality (RR: 0.95, 95% CI 0.82 to 1.12; *I*^2^ = 5%; *n* = 4 studies) (Supplementary Materials Figure S3). Moreover, high adherence to a Mediterranean dietary pattern was inversely associated with risk of colorectal cancer (RR_observational_: 0.82, 95% CI 0.75 to 0.88; *I*^2^ = 73%, *n* = 11 studies; RR_cohort_: 0.86, 95% CI 0.80 to 0.92, *I*^2^ = 28%, *n* = 6 studies; RR_case-control_: 0.71, 95% CI 0.57 to 0.88, *I*^2^ = 88%, *n* = 5 studies) (Supplementary Materials Figure S4) and breast cancer (RR_RCT_: 0.43, 95% CI 0.21 to 0.88, *I*^2^ = NA%, *n* = 1 study; RR_observational_: 0.92, 95% CI 0.89 to 0.96, *I*^2^ = 8%, *n* = 16 studies; RR_cohort_: 0.94, 95% CI 0.90 to 0.99, *I*^2^ = 11%, *n* = 7 studies; RR_case-control_: 0.89, 95% CI 0.85 to 0.94, I^2^ = 0%, *n* = 9 studies) (Supplementary Materials Figure S5). With respect to incidence of all other different types of cancer, results are summarized in Table 2 and the corresponding forest plots are given in Figures S2–S20.

### 3.3. Food Group Components of the MedD and Risk of Cancer

**Figure 1:**
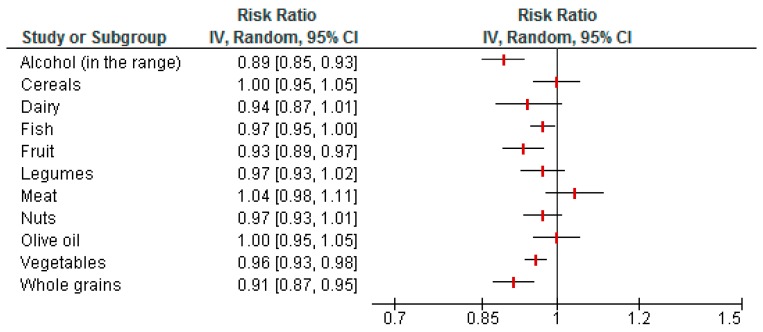
Pooled risk ratios of individual Mediterranean diet components and overall cancer risk.

Pooled estimates for the single components of the Mediterranean dietary pattern shown in Figure 1 revealed an inverse association for fruit consumption (RR: 0.93, 95% CI 0.89 to 0.97, *I*^2^ = 60%, *n* = 13 studies), vegetable intake (RR: 0.96, 95% CI 0.93 to 0.98, *I*^2^ = 0%, *n* = 14 studies), whole grain intake (RR: 0.91, 95% CI 0.87 to 0.95, *I*^2^ = 31%, *n* = 9 studies), and moderate alcohol consumption (within the range) (RR: 0.89, 95% CI 0.85 to 0.93), compared to higher intakes and cancer risk. No significant associations were observed for intakes of cereals, dairy, fish, legumes, meat, nuts, or olive oil (Figure 1).

### 3.4. Publication Bias

The Egger’s linear regression tests provided no evidence for a publication bias for overall cancer mortality (*p* = 0.86), breast cancer (*p* = 0.39), and colorectal cancer (*p* = 0.14), following comparison of the highest vs. lowest adherence to MedD category (Supplementary Materials Figures S21–S23). Funnel plots were only generated when at least 10 studies were available for a comparison. The funnel plots for risk of overall cancer mortality as well as risk of breast and colorectal cancer indicate moderate asymmetry, suggesting that publication bias cannot be completely excluded as a factor of influence on the present meta-analysis (Supplementary Materials Figures S21–S23).

## 4. Discussion

Following the synthesis of data from RCTs as well as cohort and case-control studies in the present systematic review, strongest adherence to a MedD was inversely associated with cancer mortality and risk of colorectal, breast, gastric, liver, head and neck, gallbladder, and biliary tract cancer. No significant associations could be observed with respect to other types of cancer and for cancer survivors. Considering dietary patterns instead of single nutrients or foods appears to have advantages in every respect. Foods are not consumed in separation and their health-related effects are additive or even synergistic [125]. Consequently, the Greek cohort of the EPIC study revealed that the inverse association between adherence to a MedD and cancer risk is not due to any single component of this diet but rather an effect of the complete pattern [126]. Contradictory to these observations, we could show significant inverse correlations between specific food groups typical for a MedD pattern (i.e., alcohol in moderate amounts, fruits, vegetables, and whole grains) and overall cancer risk (see Figure 1). In addition, a trend for potentially beneficial effects was detected for dairy, fish, and nuts, while meat pointed towards deleterious mechanisms. Fruits and vegetables contain numerous components known to have favorable effects on inflammatory, cellular redox, as well as metabolic processes and endothelial function, which might add up to their tumor-protective impact [127,128]. In addition, regular consumption of fruits and vegetables facilitates weight management in overweight subjects to counter obesity as a risk factor for cancer [129]. Whole grain products contain phytic acid, resistant starch, and soluble fiber, which are able to bind and neutralize potentially carcinogenic compounds in food [130]. An inverse association between whole grain intake and cancer risk was described in other meta-analyses as well [131,132]. Dairy products are included in the MedD pattern as a detrimental component. However, evidence regarding their health effects, especially for cancer, is controversial. A recent meta-analysis suggested dairy products are associated with a reduced risk for colorectal cancer risk [133], whereas a higher risk of prostate cancer has been reported [134]. Moreover, consumption of fermented dairy products was associated with a decreased risk to become obese or diabetic [135,136]. The health-promoting effects of dairy products might be explained by their high content of calcium, vitamins, and protein. Inverse associations between fish intake and all-cause as well as cancer mortality could be observed in various meta-analyses [137,138,139,140]. With respect to mechanisms, growth inhibitory, pro-apoptotic or anti-angiogenic effects can be attributed to the high amount of *n*-3 fatty acids present in fish [141,142]. Tree nuts contain anti-inflammatory, anti-oxidative, and endothelium-protective substances such as fiber, mono- and polyunsaturated fatty acids, or secondary plant metabolites which might prevent tumor pathogenesis and progression [143]. A significant cancer-protective effect of nuts could be demonstrated in a recent meta-analysis by Aune et al. [144]. Unfavorable effects of (red) meat and processed meat products might be due to ingredients promoting pro-inflammatory and pro-oxidative metabolic processes, e.g., nitrosamines, iron, or saturated fatty acids. A positive association between consumption of red meat and all-cause mortality could be observed in prospective cohorts from Europe and the US [145,146]. 

A specific and controversial “ingredient” of a MedD is ethanol, usually represented in the form of red wine. In some of the assessment indices mentioned, the highest rating/level of adherence could only be achieved when alcohol is consumed on a daily basis in moderate amounts. Ethanol is considered as a carcinogen in humans by the Agency for Research on Cancer [147]. The WCRF report summarizes alcoholic beverages as a risk factor in the development of different carcinomas (e.g., mouth, pharynx, larynx, esophagus, liver, colorectal, breast in pre- and post-menopause), with grades of evidence ranging between “convincing” and “probable”, respectively [129]. In some studies, consumption of alcoholic beverages even in low to moderate amounts was associated with an increased risk of colorectal, liver, and breast cancer as well as carcinomas of the larynx, pharynx, and esophagus [148]. Although red wine contains a number of potential protective ingredients such as anti-oxidative polyphenols, its beneficial role regarding malignant diseases is discussed controversially, e.g., with respect to non-Hodgkin’s lymphoma, renal cell carcinoma, breast cancer, or thyroid cancer [149,150,151,152]. When assessing the effects of a MedD on health and disease, one should keep in mind that the different scores for this dietary pattern may include alcoholic beverages. However, adherence to a MedD is usually defined by categories classified according to pre-defined cut off points. Thus, individuals in the highest categories cannot be discriminated according to their consumption of alcoholic beverages. Therefore, at present, it seems to be impossible to make an unequivocal statement on the effects of alcohol consumption regarding tumor pathogenesis. It seems pointless to recommend moderate alcohol consumption to hitherto abstinent individuals, especially cancer survivors. 

Taken together, these data provide evidence that the combination of food groups in order to compose a pattern corresponding to a MedD exerts benefits with respect to tumor pathogenesis. However, it remains difficult to define the MedD as a distinct pattern. According to Simopoulos, the term “Mediterranean diet” is even misleading, since there is no clear-cut definition of a MedD but rather a number of variations based on different cultural, religious, ethnical, and economical versions of this regimen practiced by the populations of countries bordering the Mediterranean Sea [153]. Frequently used indices to assess the adherence to a MedD are the ones by Trichopoulou [36], the modified MedD index by the same group [154], the alternate Mediterranean Diet index [37], or the relative Mediterranean Diet index [85]. The initial three indices chose gender-specific medians of intake of relevant food groups as cutoff-points. It seems obvious that cutoff-points defined by medians vary between different populations, leading to an almost indefinite number of MedD subtypes. As a result, it will become impossible to differentiate between preferable and dissenting intakes [155]. Moreover, determination of the components of a MedD is inconsistent between different epidemiological studies. The characteristics of a MedD have changed considerably since the 1960s. Taking into account only some of its constituents does not justify categorization as a MedD pattern [156]. Thus, assessing the components of a MedD with respect to their potential tumor-preventive properties is subject to a number of prior conditions. It has to be noted that a large number of studies enrolled in the present systematic review fulfill these requirements, such as the special consideration of whole-grain products in the cereals category [37,74,104,110,111,112,113] or of extra virgin olive oil as the predominant provider of fat [85]. On the other hand, the protective effects of olive oil are most likely due to its content of polyphenols as well as their interactions [157]. Because of manufacturing conditions, not all of these compounds are present in every brand of olive oil, not even in extra virgin olive oil varieties [158]. Additionally, most studies measured intake of alcoholic beverages without special consideration of red wine (exceptions, e.g., [74,113]). For future assessments of a MedD it might be helpful to use a literature-based score implementing portions of each food group to achieve a pre-defined score such as the one suggested by Sofi et al. [30]. In addition, it might be helpful to explore linear trends via dose-response analyses as well as possible non-linear associations as has recently been done for the relation between 12 food groups and risk of type 2 diabetes mellitus [136].

Most of the data synthesized meta-analytically in the present systematic review were derived from epidemiological studies performed in either the US or European countries. These countries differ regarding their respective data on cancer prevalence, while at the same time, data from other countries are missing [159]. Additional to differences in adherence to varying dietary patterns, country-specific data may be explained by genetic factors, geographic features, or mixed exposures to carcinogens. The different manifestation of harmful and beneficial determinants in single countries might contribute to both over- as well as underestimation of the effects of a MedD. In some studies, the inverse association between adherence to a MedD and risk of tobacco-related cancers was significantly more pronounced in the subgroup of active smokers [78,160].

The validity of our results is further limited by methodological shortcomings of some of the studies enrolled in the present systematic review. The most common tool to assess a dietary regimen in observational studies is the food frequency questionnaire. It is an established and cost-effective instrument, however, with a number of disadvantages. Lesser number of items and short survey periods might not necessarily represent a dietary pattern with impact on chronic diseases and mortality. However, most of the included studies did assess the whole diet via FFQ over a period of more than 12 months.

Another limitation was the lopsided availability of studies by type of cancer. The most frequently investigated tumor localizations in the present study were breast, colorectal, prostate, and head-and-neck cancer, while other types of cancer such as lung or esophageal cancer were explored to a lesser extent. Taken together, these limitations might at least in part explain the considerable heterogeneity present in our systematic review (Table 2).

The observed high statistical heterogeneity for cancer mortality was related to the different definitions for MedD pattern. Additional analyses indicated lower levels of heterogeneity when studies were grouped according to similar definitions of a MedD pattern (e.g., alternate MedD, or traditional MedD). The high levels of heterogeneity observed for colorectal cancer risk in the main analysis could not be confirmed for cohort studies in the stratified analysis, and were mainly driven by one large case-control study.

Due to the low number of studies available for several cancer types, we were not able to investigate properly potential sources of heterogeneity. However, statistical heterogeneity might be related to differences in adjustment factors, as well as heterogeneous definition of the MedD.

To some degree, our results might depend on residual confounders. Hypothesis-Driven dietary patterns [161] represent one aspect of a multidimensional lifestyle complex including other factors such as exercise, smoking behavior, and alcohol consumption [162]. Although the results of the studies are most often adjusted for these factors via multivariate analyses, we cannot exclude potential confounders as another limitation affecting our results. The benefits of a MedD were found to be depending on cohort characteristics such as smoking behavior [85], age groups [37,85], hormonal status [37,116], or gender [89,94].

Strengths of the present study are the extensive literature research with strict adherence to quality standards set by PRISMA guidelines. Data were synthesized predominantly from observational studies enrolling a high number of participants, which, in some cases, may compensate for the low number of studies investigating specific tumor localizations.

## 5. Conclusions

In conclusion, the present update of our systematic review and meta-analyses provided additional important evidence for a beneficial effect of high adherence to MedD with respect to primary prevention overall cancer risk and specific types of cancer, especially colorectal cancer. These observed beneficial effects are mainly driven by higher intakes of fruits, vegetables, and whole grains. Moreover, we report for the first time a small decrease in breast cancer risk (6%) with the pooling seven cohort studies. To further elucidate the relationship between Mediterranean dietary patterns and cancer types, future studies should adopt a precise definition of a MedD.

## Figures and Tables

**Table 1:** General study characteristics of included studies (randomized controlled trials, cohort and case-control studies).

Author	Country Study Name	Study Design	Outcome	Population Follow-Up (Years)	Age at Entry	Sex	Components of Score Score Range	Adjustment	RR/HR/OR (95% CI) Multivariable Adjusted
De Lorgeril et al. [32]	France Lyon Diet Heart Study	RCT	Cancer mortality Lung cancer Digestive tract cancer Urinary tract cancer Throat cancer	605 4	54	M/W	1. MedD: More bread, more root vegetables and green vegetables, more fish, less meat, no day without fruit, and butter and cream to be replaced with margarine supplied by the study; supplemented with a rapeseed oil-based margarine 2. control diet	NA	Cancer mortality MedD with margarine RR: 0.75 (0.17, 3.33) versus control diet Lung cancer MedD with margarine RR: 2.01 (0.37, 10.87) versus control diet Throat cancer MedD with margarine RR: 0.14 (0.01, 2.76) versus control diet
Toledo et al. [33]	Spain PREDIMED	RCT	Breast cancer	4282 4.8	60–80	W	1. MedD supplemented with extra-virgin olive oil; 2. MedD supplemented with mixed nuts; 3. control diet (advice to reduce dietary fat)	Age, study site, BMI, waist-to-height ratio, hormone therapy, PA, total energy intake, alcohol, age at menopause, baseline adherence to the MedD	MedD with olive oil RR: 0.32 (0.13, 0.79) versus control diet MedD with nuts RR: 0.59 (0.26, 1.35) versus control diet Both MedD RR: 0.43 (0.21, 0.88) versus control diet
Anic et al. [50]	United States NIH-AARP	Cohort	Lung cancer	460,770 10.5	50–71	M/W	1.↑ whole grains; 2.↑ vegetables; 3.↑ fruits; 4.↑ nuts; 5.↑ legumes; 6.↑ fish; 7.↑ MUFA:SFA ratio; 8.↓ red and processed meats; 9.↔ alcohol MedD score range: 0–9	Age, sex, race, education, BMI, PA, total energy, smoking status, cigarettes per day, time since quitting smoking, and regular use of cigars/pipes	HR: 0.85 (0.79, 0.91) for fifth versus first quintile
Butler et al. [64]	Singapore SCHS	Cohort	Breast cancer	34,028 >5	45–74	W	1.↑ cereals; 2.↑ vegetables; 3.↑ fruits/nuts; 4.↑ legumes; 5.↑ fish; 6.↑ MUFA: SFA ratio; 7.↓ meat; 8.↓ dairy; 9.↓ carbohydrates; 10. ↓ alcohol MedD score range: 0–10	NA	HR: 0.96 (0.76, 1.21) for third versus first tertile
Dugué et al. [51]	Australia MCCS	Cohort	Urothelial cell carcinoma	37,442 21.3	40–69	M/W	1.↑ vegetables; 2.↑ fruits; 3.↑ cereals; 4.↑ legumes; 5.↑ fish; 6.↑ olive oil; 7.↓ dairy; 8.↓ red meat; 9.↔ alcohol MedD score range: 0–9	Sex, country of birth, smoking, alcohol, BMI, PA, education, socioeconomic status	HR: 0.89 (0.62, 1.26) for fifth versus first quintile
Haridass et al. [52]	United States CTS	Cohort	Breast cancer	90,244 16	22–104	W	1.↑ vegetables; 2.↑ fruits; 3.↑ nuts/legumes; 4.↑ fish; 5.↑ whole grains; 6.↑ MUFA:SFA; 7.↓ red and processed meats; 8.↔ alcohol MedD score range: 0–8	Age at baseline, race, menopausal status, age at menarche, breast cancer family history, smoking, BMI, energy intake	HR: 0.91 (0.83, 1.01) for fifth versus first quintile
Hirko et al. [53]	United States NHS	Cohort	Breast cancer (by molecular subtype)	100,643 22	30–55	W	1.↑ fruits; 2.↑ vegetables; 3.↑ legumes and soy; 4.↑ nuts; 5.↑ fish and seafood; 6.↑ whole grains; 7.↑ MUFA:SFA; 8.↓ red and processed meat; 9.↔ alcohol MedD score range: 0–9	BMI at age 18, weight change since age 18, PA, energy intake, parity/age at first birth, menopausal hormone use, oral contraceptive use, age at menarche, age at menopause, family history of breast cancer, benign breast cancer diagnosis	Luminal A HR: 1.09 (0.91, 1.30) for fifth quintile (5.5–9.0) versus first (0–2.6) quintile Luminal B HR: 1.02 (0.76, 1.37) for fifth quintile (5.5–9.0) versus first (0–2.6) quintile HER2 type HR: 0.74 (0.42, 1.29) for fifth quintile (5.5–9.0) versus first (0–2.6) quintile Basal-like HR: 0.78 (0.49, 1.26) for fifth quintile (5.5–9.0) versus first (0–2.6) quintile Unclassified HR: 0.89 (0.41, 1.89) for fifth quintile (5.5–9.0) versus first (0–2.6) quintile
Hodge et al. [54]	Australia MCCS	Cohort	Lung cancer	35,303 18	40–69	M/W	1.↑ vegetables; 2.↑ fruits; 3.↑ cereals; 4.↑ legumes; 5.↑ fish; 6.↑ olive oil; 7.↓ dairy; 8.↓ red meat; 9.↔ alcohol MedD score range: 0–9	Pack-years, years since quitting smoking, smoking status, country of birth, education, BMI, PA, sex, SEIFA quintile, energy	HR: 0.64 (0.45, 0.90) for highest category (7–9) versus lowest category (0–3)
Jacobs et al. [55]	United States MEC	Cohort	Colorectal cancer mortality among cancer survivors	4204 6.0	45–75	M/W	1.↑ vegetables; 2.↑ fruits; 3.↑ nuts; 4.↑ legumes; 5.↑ fish; 6.↑ whole grains; 7.↑ MUFA:SFA; 8.↓ red and processed meat; 9.↔ alcohol MedD score range: 0–9	Age at diagnosis, ethnicity, stage at diagnosis, total energy intake, smoking status, pack-years, PA, education, radiation treatment, chemotherapy, NSAID use, family history of CRC, comorbidities	Men HR: 1.07 (0.81, 1.42) for fourth quartile (6–9) versus first quartile (0–2) Women HR: 0.74 (0.54, 1.01) for fourth quartile (6–9) versus first quartile (0–2)
Jones et al. [65]	United Kingdom WHS	Cohort	Colorectal cancer	35,372 17.4	35–69	W	1.↑ vegetables; 2.↑ fruits & nuts; 3.↑ legumes; 4.↑ cereals; 5.↑ fish; 6.↑ MUFA+PUFA: SFA; 7.↓ dairy; 8.↓ meat; 9. ↓ poultry; 10.↔ alcohol MedD score range: 0–10	Age, BMI, energy intake, physical activity, smoking status, socioeconomic status and family history of colorectal cancer	HR: 0.82 (0.57, 1.17) Fifth quintile (7–10) versus first quintile (0–2)
Larsson et al. [56]	Sweden Swedish Mammography Cohort Cohort of Swedish Men	Cohort	Biliary tract cancer Gallbladder cancer	76,014 13.3	45–83	M/W	1.↑ vegetables; 2.↑ fruits; 3.↑ legumes and nuts; 4.↑ whole-grains; 5.↑ fish; 6.↓ full-fat dairy products; 7.↓ red meat and processed meat; 8.↑ olive oil; 9.↔ alcohol MedD score range: 9–45	Age, sex, education, smoking status and pack-years of smoking, diabetes, BMI, total energy intake	Extrahepatic Biliary tract cancer HR: 0.41 (0.25, 0.67) for third tertile (29–45) to first tertile (9–24) Gallbladder cancer HR: 0.42 (0.23, 0.79) for third tertile (29–45) to first tertile (9–24) Intrahepatic BTC HR: 0.71 (0.25, 2.04) for third tertile (29–45) to first tertile (9–24)
Lassale et al. [57]	Europe EPIC	Cohort	Cancer mortality	451,256 12.8	25–70	M/W	MedD: 1. fruits; 2. vegetables; 3. legumes; 4. grains; 5. fish; 6. meat; 7. dairy products; 8. MUFA:SFA; 9. alcohol MedD score range: 0–9 rMedD: 1. fruits; 2. vegetables; 3. legumes; 4. grains; 5. fish; 6. meat; 7. dairy products; 8. olive oil; 9. alcohol rMED score range: 0–18 MSDPS: 1. whole grain cereals (8 servings/day); 2. fruits (3 servings/day); 3. vegetables (6 servings/day); 4. dairy (2 servings/day); 5. fish (6 servings/week); 6. wine (3 and 1.5 servings/day for men and women, respectively); 7. poultry (4 servings/week); 8. olives, legumes, and nuts (4 servings/week); 9. potatoes (3 servings/week); 10. eggs (3 servings/week); 11. sweets (3 servings/week); 12. meats (1 servings/week); 13. olive oil (exclusive use) MSDPS score range: 0–100	Age at baseline, BMI, PA, smoking status, education, stratified by sex and study center	MedD HR: 0.82 (0.77, 0.88) for fourth versus first quartile rMED HR: 0.82 (0.77, 0.88) for fourth versus first quartile MSDPS HR: 0.84 (0.79, 0.90) for fourth versus first quartile
Maisonneuve et al. [58]	Italy COSMOS	Cohort	Lung cancer	4336 8.5	50–84	M/W	1.↑ vegetables; 2.↑ fruits; 3.↑ nuts; 4.↑ cereals; 5.↑ legumes; 6.↑ fish; 7.↓ red and processed meats; 8.↔ alcohol; 9.↑ MUFA:SFA MedD score range: 0–9	Baseline risk probability (age, sex, smoking duration, smoking intensity, years of smoking cessation, asbestos exposure), total energy, dietary inflammatory index	HR: 0.20 (0.04, 0.91) for highest score category (8–9) versus lowest score category (0–1)
Molina-Montes et al. [59]	Europe EPIC	Cohort	Exocrine pancreatic cancer	477,309 11.3	35–70	M/W	1.↑ fruits and nuts; 2.↑ vegetables; 3.↑ legumes; 4.↑ fish and seafood; 5.↑ olive oil; 6.↑ cereals; 7.↓ meat; 8.↓ dairy products MedD score range: 0–16	Total energy intake, BMI, smoking status and intensity, alcohol intake, diabetes, stratified by age, sex and study centre	HR: 0.99 (0.77, 1.26) for highest score category (10–16) versus lowest score category (0–5)
Park et al. [60]	United States MEC	Cohort	Colorectal cancer	190,949 16	45–75	M/W	1.↑ vegetables; 2.↑ fruits; 3.↑ nuts; 4.↑ legumes; 5.↑ fish; 6.↑ whole grains; 7.↑ MUFA:SFA; 8.↓ red and processed meat; 9.↔ alcohol MedD score range: 0–9	Age at cohort entry, family history of colorectal cancer, history of colorectal polyp, BMI, smoking, multivitamin, nonsteroidal anti-inflammatory drugs, physical activity, menopausal status, menopausal hormone therapy use for women only, and total energy intake	HR: ♂ 0.84 (0.72, 0.97) HR: ♀ 0.96 (0.82, 1.13) for highest score category (6–9) versus lowest score category (0–2)
Van den Brandt et al. [61]	The Netherlands NCS	Cohort	Breast cancer	62,573	55–69	W	1.↑ vegetables; 2.↑ fruits; 3.↑ nuts; 4.↑ whole grains; 5.↑ legumes; 6.↑ fish; 7.↑ MUFA:SFA; 8.↓ red and processed meats; 9.↔ alcohol MedD score range: 0–9	Age, smoking, duration, body height, BMI, non-occupational physical activity, highest level of education, family history of breast cancer in mother or sisters, history of benign breast disease, age at menarche, parity, age at first birth, age at menopause, oral contraceptive use, postmenopausal HRT, energy intake and alcohol intake	HR: 0.87 (0.72, 1.06) for highest score category (6–8) vs. lowest score category (0–3) Without alcohol component
Vargas et al. [62]	United States WHIOS	Cohort	Colorectal cancer	78,273 12.4	50–79	W	1.↑ vegetables; 2.↑ fruits; 3.↑ nuts; 4.↑ whole grains; 5.↑ legumes; 6.↑ fish; 7.↑ MUFA:SFA; 8.↓ red and processed meats; 9.↔ alcohol MedD score range: 0–9	Age, race/ethnicity, PA, education, smoking, hormone replacement therapy	HR: 0.91 (0.74, 1.11) for fifth quintile (6–9) versus first quintile (0–2)
Whalen et al. [63]	United States REGARDS	Cohort	Cancer mortality	21,423 6.25	>45	M/W	1.↑ vegetables; 2.↑ fruits; 3.↑ lean meats; 4.↑ fish; 5.↑ nuts; 6.↑ MUFA:SFA; 7.↓ red and processed meats; 8.↓ sodium; 9. ↔ dairy; 10.↔ grains and starches; 11.↔ alcohol MedD score range: 11–55	Sex, race, total energy intake, BMI, PA, smoking, annual income, hormone replacement therapy use (in women) at baseline	HR: 0.64 (0.48, 0.84) for fifth versus first quintile
**Author**	**Country Study Name**	**Study Design**	**Outcome**	**Cases/Controls**	**Age at Entry**	**Sex**	**Components of Score** **Score Range**	**Adjustment**	**Multivariable Adjusted**
Askari et al. [66]	Iran	Case-control	Prostate cancer	52/104	40–78	M	1. whole grain cereals (8 servings/day); 2. fruits (3 servings/day); 3. vegetables (6 servings/day); 4. dairy products (2 servings/day); 5. fish and other seafood (6 servings/week); 6. poultry (4 servings/week); 7. olives/legumes/nuts (4 servings/week); 8. potatoes and other starchy roots (3 servings/week); 9. eggs (3 servings/week); 10. sweets (3 servings/week); 11. meat (1 servings/week); 12. olive oil (exclusive use) MedD score range: 0–100	Age, BMI, smoking, energy intake, education, diabetes	OR: 0.28 (0.08, 0.91) for third versus first tertile
Campagna et al. [67]	Italy	Case-control	Lymphoma	322/446	n.d	M/W	1.↑ fruits; 2.↑ vegetables; 3.↑ legumes; 4.↑ fresh fish and seafood; 5.↑ pasta, rice, and bread; 6.↓ red meat; 7.↔ wine MedD score range: n.d	Age, sex, education	OR: 0.9 (0.6, 1.5) for fifth versus first quintile
Castello et al. [43]	Spain	Case-control	Breast cancer	1181/1682	20–85	W	PCA: ↑ fish; ↑ vegetables; ↑ legumes; ↑ boiled potatoes; ↑ fruits; ↑ olives and vegetable oil; ↓ juices	menopausal status, age, education, BMI, age at first delivery, family history of breast cancer, physical activity, smoking status, caloric intake and alcohol intake as fixed effects and province of residence as a random effect term	OR: 0.90 (0.69, 1.17) for fourth vs. first quartile
Castello et al. [44]	Spain	Case-control	Prostate cancer	754/1277	38–85	M	PCA: ↑ fish; ↑ vegetables; ↑ legumes; ↑ boiled potatoes; ↑ fruits; ↑ olives and vegetable oil; ↓ juices	Age, education, BMI, age at first delivery, family history of prostate cancer and caloric intake as fixed effects and province of residence as a random effect	OR: 0.91 (0.66, 1.25) for fourth vs. first quartile
Giraldi et al. [68]	Italy	Case-control	Head and neck cancer	500/433	n.d	M/W	1.↑ fruits; 2.↑ vegetables; 3.↑ legumes; 4.↑ fish; 5.↓ meat and meat products; 6.↔ alcohol MedD score range: 0–12	Age, sex, smoking, alcohol, total energy intake	OR: 0.64 (0.58, 0.71) per 1-point increase
Rosato et al. [69]	Italy	Case-control	Colorectal cancer	3745/6804	19–74	M/W	1.↑ vegetables; 2.↑ legumes; 3.↑ fruits and nuts; 4.↑ cereals; 5.↑ fish and seafood; 6.↑ MUFA:SFA; 7.↓ dairy; 8.↓ meat and meat products; 9.↔ alcohol MedD score range: 0–9	Age, sex, calendar period, center, education, BMI, PA, family history of intestinal cancer, total energy intake	OR: 0.52 (0.43, 0.62) for highest score category (7–9) versus lowest score category (0–2)
Stojanovic 2017 [70]	Italy	Case-control	Gastric cancer	223/223	NA	NA	NA	NA	OR: 0.70 (0.61, 0.81)
Turati et al. [71]	Italy	Case-control	Nasopharyngeal cancer	198/594	18–76	M/W	1.↑ vegetables; 2.↑ legumes; 3.↑ fruits and nuts; 4.↑ cereals; 5.↑ fish and seafood; 6.↑ MUFA:SFA; 7.↓ dairy products; 8.↓ meats; 9.↔ alcohol MedD score range: 0–9	sex, age, place of residence, education, smoking, total energy intake	OR: 0.66 (0.44, 0.99) for highest score category (>6) versus lowest score category (0–4)
Wang et al. [72]	China	Case-control	Nasopharyngeal cancer	600/600		M/W	1.↑ whole grains; 2.↑ vegetables; 3.↑ fruits; 4.↑ legumes; 5.↑ nuts; 6.↑ fish; 7.↑ MUFA:SFA; 8.↔ alcohol; 9.↓ red and processed meats MedD score range: 0–9	Age, BMI, occupation, marital status, education, household income, smoking, drinking, exposure to potential toxic substances, multivitamin supplements, chronic rhinitis history, PA, energy intake, preserved vegetables and animal food	OR: 0.85 (0.59, 1.22) for fourth versus first quartile

BMI, Body Mass Index; HR, Hazard ratio; M, men; MedD, Mediterranean Diet; MSDPS, Mediterranean Style Dietary Pattern Score; MUFA, monounsaturated fat; NA, not applicable; OR, Odds ratio; PA, physical activity; PCA, principal component analysis; PUFA, polyunsaturated fat; RCT, randomized controlled trials; rMedD, relative Mediterranean diet score; RR, Risk ratio; SFA, saturated fatty acids; W, women.

**Table 2:** Risk ratio/odds ratio associated with the highest adherence to Mediterranean dietary pattern.

Outcome	No. of Studies	Study Type	Risk Ratio/Odds Ratio	95% CI	*I*^2^ (%)
Cancer mortality	1	RCT	0.75	0.17, 3.33	NA
	14	Cohort	0.86	0.81, 0.91	82
Colorectal cancer	11	Observational	0.82	0.75, 0.88	73
incidence	6	Cohort	0.86	0.80, 0.92	28
	5	Case-control	0.71	0.57, 0.88	88
Breast cancer	1	RCT	0.43	0.21, 0.88	NA
	16	Observational	0.92	0.89, 0.96	8
incidence	7	Cohort	0.94	0.90, 0.99	11
	9	Case-control	0.89	0.85, 0.94	0
Prostate cancer	6	Combined	0.96	0.92, 1.00	0
incidence	3	Cohort	0.96	0.92, 1.00	0
	3	Case-control	0.90	0.64, 1.26	52
Gastric cancer	4	Combined	0.72	0.60, 0.86	55
incidence	2	Cohort	0.82	0.61, 1.10	49
	2	Case-control	0.65	0.53, 0.79	53
Liver cancer	2	Combined	0.58	0.46, 0.73	0
incidence	1	Cohort	0.62	0.47, 0.82	NA
	1	Case-control	0.51	0.34, 0.77	NA
Esophageal cancer	2	Combined	0.49	0.22, 1.09	83
incidence	1	Cohort	0.68	0.34, 1.36	NA
	1	Case-control	0.26	0.13, 0.52	NA
Head and neck cancer	1	RCT	0.14	0.01, 2.76	86
	7	Observational	0.49	0.37, 0.66	87
incidence	1	Cohort	0.61	0.33, 1.14	77
	6	Case-control	0.46	0.32, 0.67	89
Endometrial cancer	3	Combined	0.72	0.40, 1.31	94
incidence	1	Cohort	0.98	0.82, 1.17	NA
	2	Case-control	0.61	0.29, 1.29	89
Respiratory cancer	1	RCT	2.01	0.37, 10.87	NA
incidence	3	Cohort	0.71	0.49, 1.02	66
Bladder cancer incidence	2	Cohort	0.85	0.72, 1.01	NA
Pancreatic cancer	2	Combined	0.69	0.34, 1.41	92
	1	Cohort	0.99	0.77, 1.27	NA
	1	Case-control	0.48	0.35, 0.66	NA
Gallbladder cancer	1	Cohort	0.42	0.23, 0.77	NA
Biliary tract cancer	1	Cohort	0.44	0.29, 0.67	NA
Ovarian cancer	1	Cohort	0.91	0.71, 1.17	NA
Lymphoma	1	Case-control	0.90	0.60, 1.35	NA
Cancer mortality among cancer survivors	4	Cohort	0.95	0.82, 1.12	5
Recurrence among cancer survivors	1	Cohort	0.61	0.18, 2.07	NA

CI, confidence interval; NA, not applicable; RCT, randomized controlled trial.

## Supplementary Materials

The following are available online at www.mdpi.com/2072-6643/9/10/1063/s1.

## Appendix A

Search strategy for PubMed: (“Mediterranean diet” OR “Mediterranean” OR “diet” OR “dietary pattern” OR “dietary score” OR “dietary adherence”) AND (“cancer” OR “neoplasm” OR “neoplastic disease” OR “survivors” OR “recurrence”) AND (“prospective” OR “follow- up” OR “cohort” OR “longitudinal”).
