# Supplementary material for: Adherence to Mediterranean Diet and Risk of Cancer: An Updated Systematic Review and Meta-Analysis
Source: Nutrients. 2017 Sep 26;9(10):1063. doi: 10.3390/nu9101063 (PMC5691680; doi:10.3390/nu9101063)
Supplement: Supplementary file 1 [file nutrients-09-01063-s001.zip › nutrients-222654-supplementary.pdf]

Supplementary Figure S1: Updated Flow chart for meta-analysis article selection process.

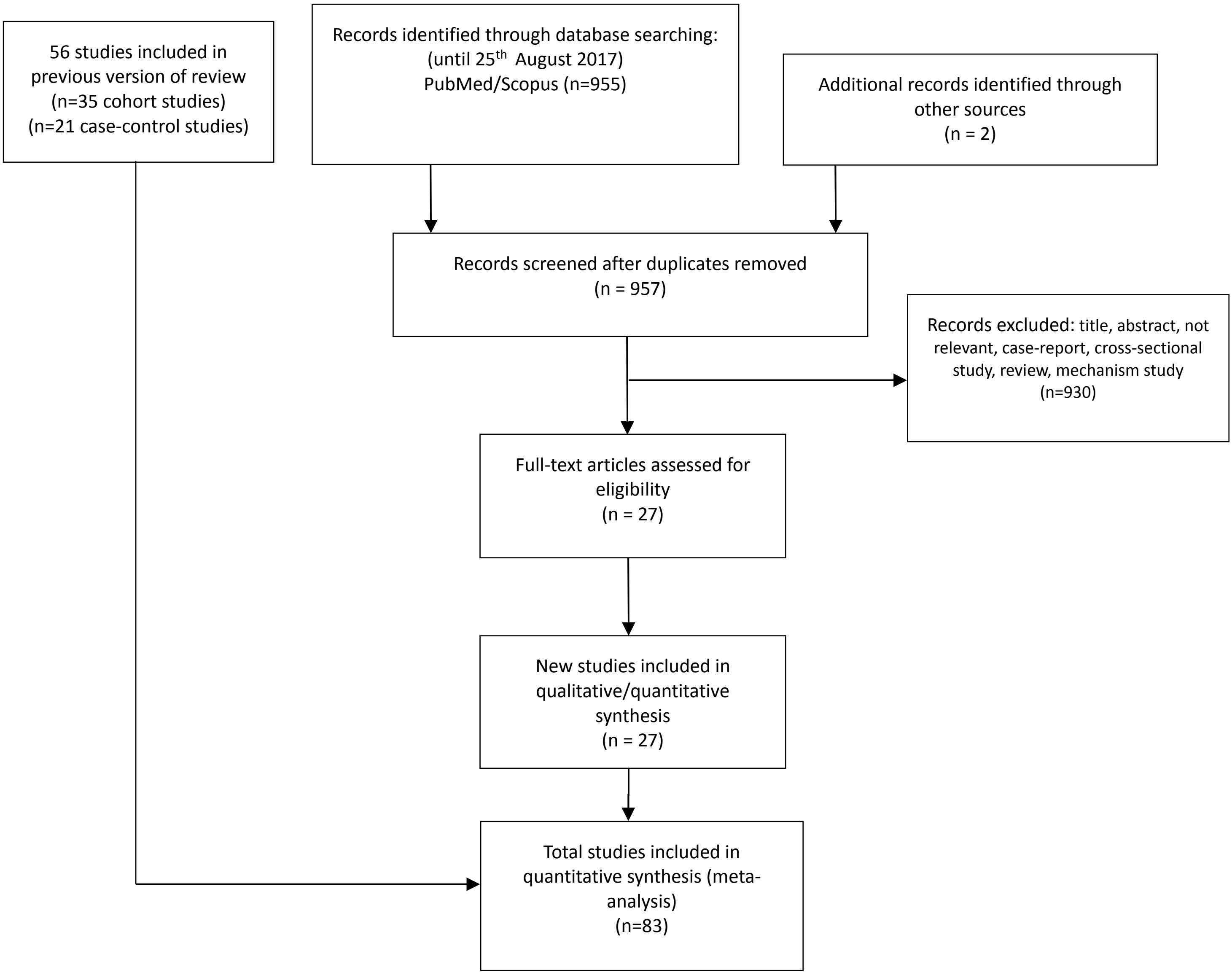

| Study or Subgroup | Weight | Risk Ratio IV, Random, 95% CI |
|---|---|---|
| **1.2.1 RCT** | | |
| de Lorgeril 1994 | 100.0% | 0.75 [0.17, 3.33] |
| **Subtotal (95% CI)** | **100.0%** | **0.75 [0.17, 3.33]** |
| Heterogeneity: Not applicable | | |
| Test for overall effect: Z = 0.37 (P = 0.71) | | |
| **1.2.2 cohort** | | |
| Cuenca-García 2014 | 0.9% | 1.63 [0.91, 2.92] |
| George 2014 | 6.5% | 0.80 [0.70, 0.91] |
| Harmon 2015 M | 8.6% | 0.81 [0.75, 0.87] |
| Harmon 2015 W | 7.7% | 0.84 [0.76, 0.93] |
| Knoops 2004 | 3.4% | 0.90 [0.70, 1.16] |
| Lagiou 2006 | 2.2% | 0.80 [0.57, 1.12] |
| Lassale 2016 | 9.1% | 0.82 [0.77, 0.87] |
| LopezGarcia 2014 HPFS | 2.2% | 0.88 [0.63, 1.23] |
| LopezGarcia 2014 NHS | 1.1% | 0.80 [0.48, 1.33] |
| Martinez-Gonzalez 2012 | 2.1% | 1.03 [0.73, 1.45] |
| Menotti 2011 | 6.7% | 0.84 [0.74, 0.95] |
| Reedy 2014 M | 9.8% | 0.80 [0.77, 0.83] |
| Reedy 2014 W | 9.0% | 0.79 [0.74, 0.84] |
| Tognon 2012 M | 9.3% | 0.92 [0.87, 0.97] |
| Tognon 2012 W | 9.1% | 0.98 [0.92, 1.04] |
| Vormund 2014 | 9.7% | 0.97 [0.93, 1.01] |
| Whalen 2017 | 2.8% | 0.64 [0.48, 0.85] |
| **Subtotal (95% CI)** | **100.0%** | **0.86 [0.81, 0.91]** |
| Heterogeneity: Tau² = 0.01; Chi² = 88.97, df = 16 (P < 0.00001); I² = 82% | | |
| Test for overall effect: Z = 5.31 (P < 0.00001) | | |
| Test for subgroup differences: Chi² = 0.03, df = 1 (P = 0.86), I² = 0% | | |

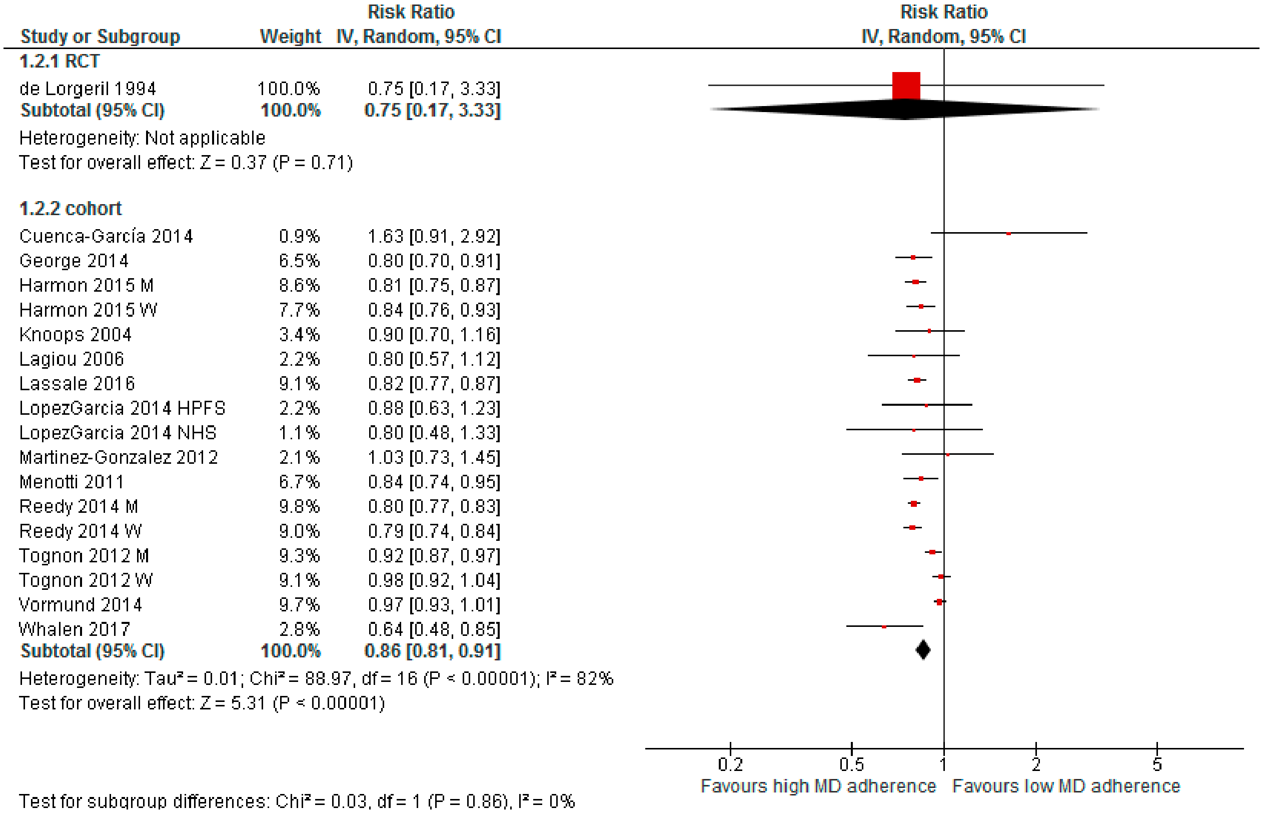


Supplementary Figure S2. Forest plot showing pooled risk ratio (RRs) with 95% CI for risk of cancer mortality for one RCT, and fourteen cohort studies.

| Study or Subgroup | Weight | Risk Ratio IV, Random, 95% CI |
|---|---|---|
| **1.1.1 Breast** | | |
| Kim 2011 | 12.3% | 1.15 [0.74, 1.79] |
| **Subtotal (95% CI)** | **12.3%** | **1.15 [0.74, 1.79]** |
| Heterogeneity: Not applicable | | |
| Test for overall effect: Z = 0.62 (P = 0.53) | | |
| **1.1.2 Colorectal** | | |
| Fung 2014 | 9.0% | 0.84 [0.50, 1.41] |
| Jacobs 2016 M | 29.4% | 1.07 [0.81, 1.41] |
| Jacobs 2016 W | 23.3% | 0.74 [0.54, 1.01] |
| **Subtotal (95% CI)** | **61.7%** | **0.89 [0.69, 1.14]** |
| Heterogeneity: Tau² = 0.02; Chi² = 3.04, df = 2 (P = 0.22); I² = 34% | | |
| Test for overall effect: Z = 0.92 (P = 0.36) | | |
| **1.1.3 Prostate** | | |
| Kenfield 2013 | 26.0% | 1.01 [0.75, 1.36] |
| **Subtotal (95% CI)** | **26.0%** | **1.01 [0.75, 1.36]** |
| Heterogeneity: Not applicable | | |
| Test for overall effect: Z = 0.07 (P = 0.95) | | |
| **Total (95% CI)** | **100.0%** | **0.95 [0.82, 1.12]** |
| Heterogeneity: Tau² = 0.00; Chi² = 4.21, df = 4 (P = 0.38); I² = 5% | | |
| Test for overall effect: Z = 0.58 (P = 0.56) | | |
| Test for subgroup differences: Chi² = 1.11, df = 2 (P = 0.58), I² = 0% | | |

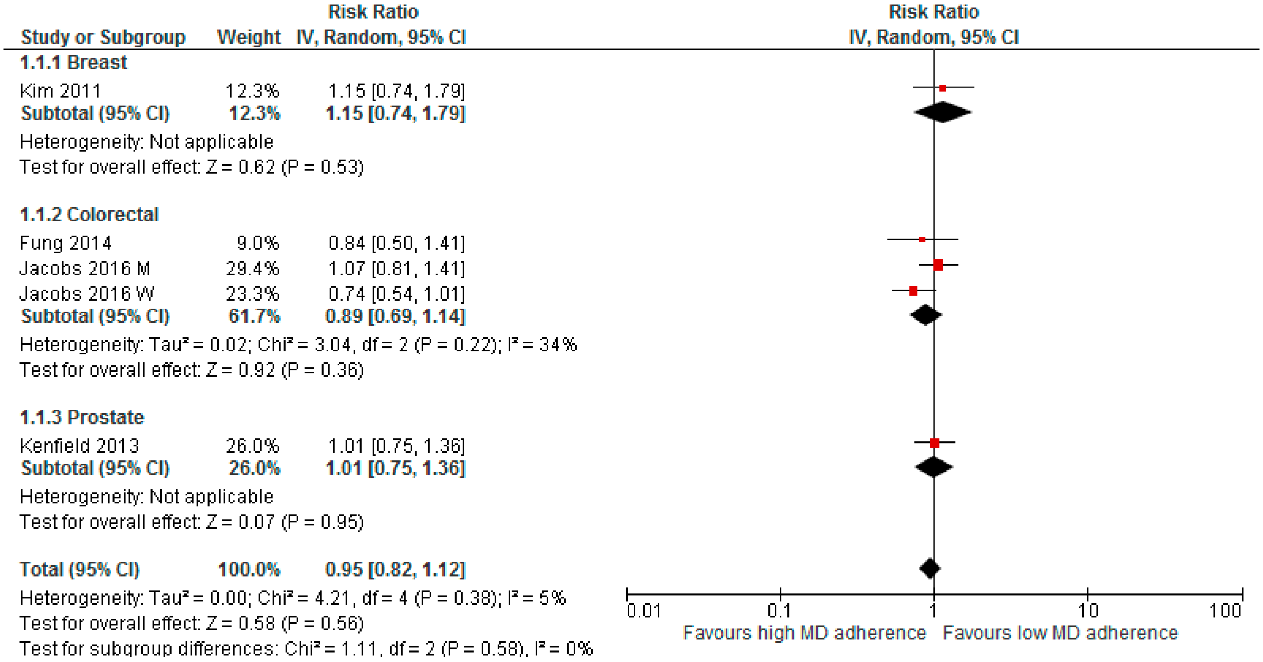


Supplementary Figure S3. Forest plot showing pooled risk ratios (RRs) with 95% CI for risk of cancer mortality among cancer survivors for four cohort studies.

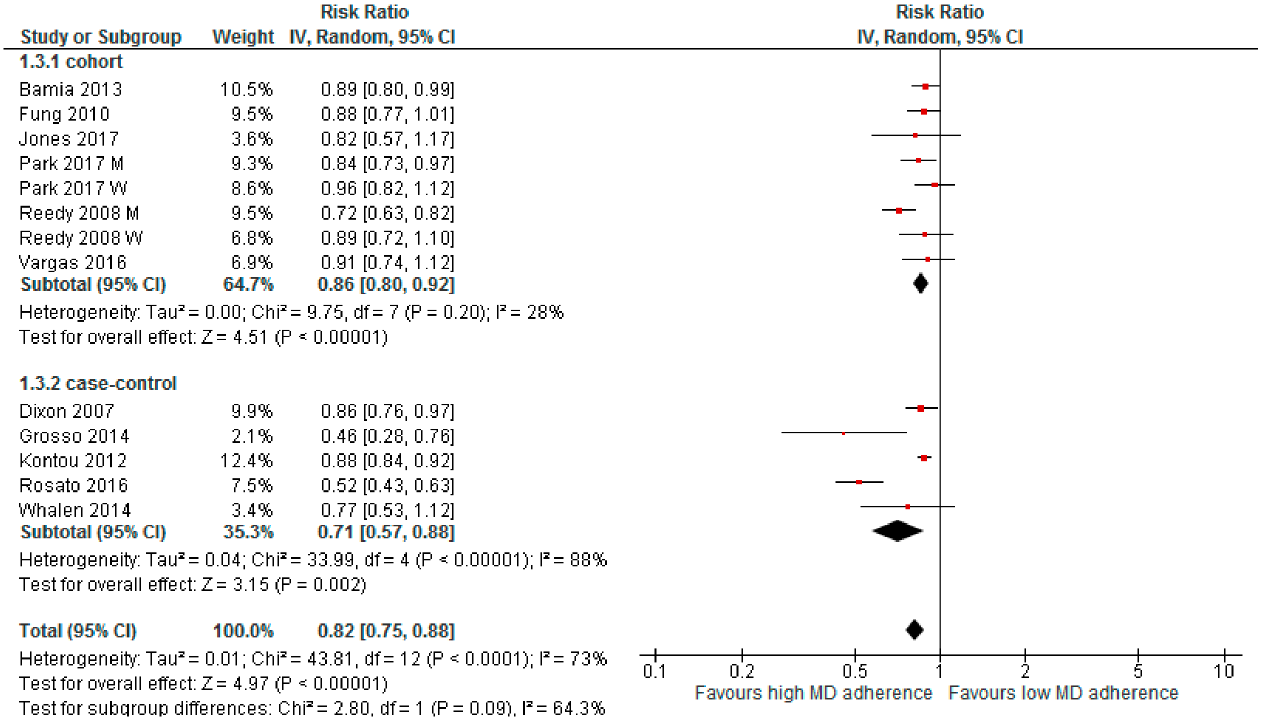


Supplementary Figure S4. Forest plot showing pooled risk ratio (RRs) with 95% CI for risk of colorectal cancer for six cohort studies, and five case-control studies.

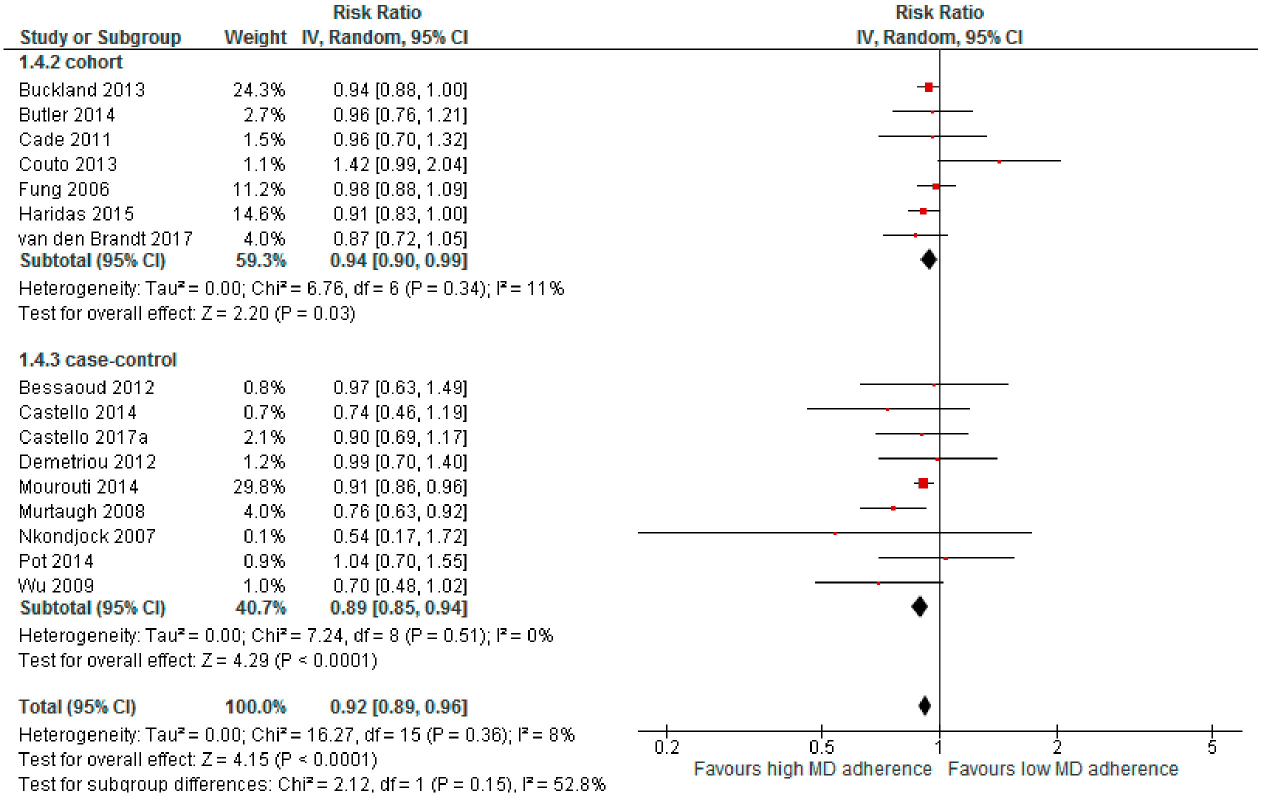


Supplementary Figure S5. Forest plot showing pooled risk ratio (RRs) with 95% CI for risk of breast cancer for seven cohort studies and nine case-control studies.

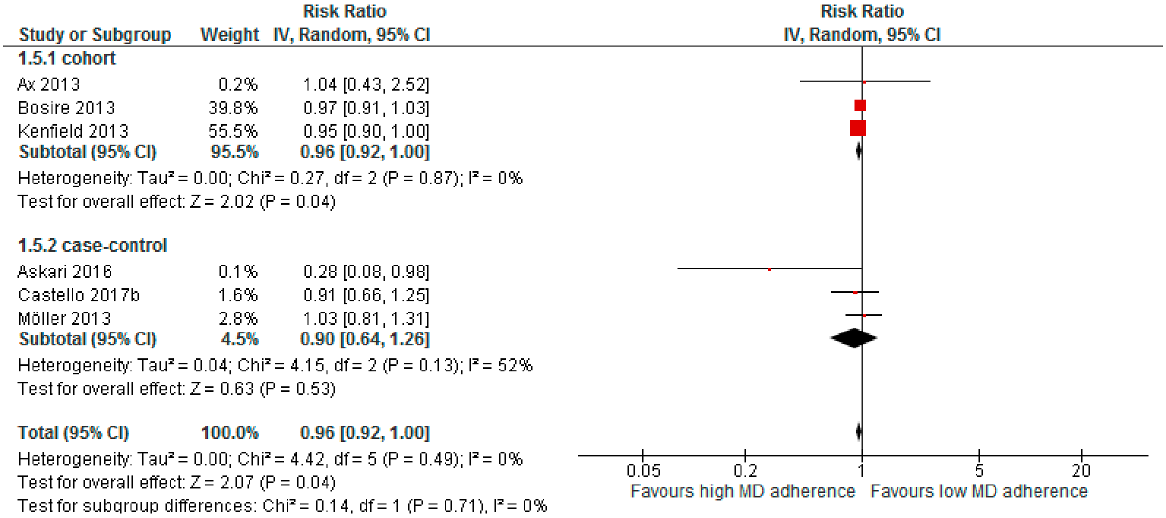


Supplementary Figure S6. Forest plot showing pooled risk ratio (RRs) with 95% CI for risk of prostate cancer for three cohort studies and three case-control studies.

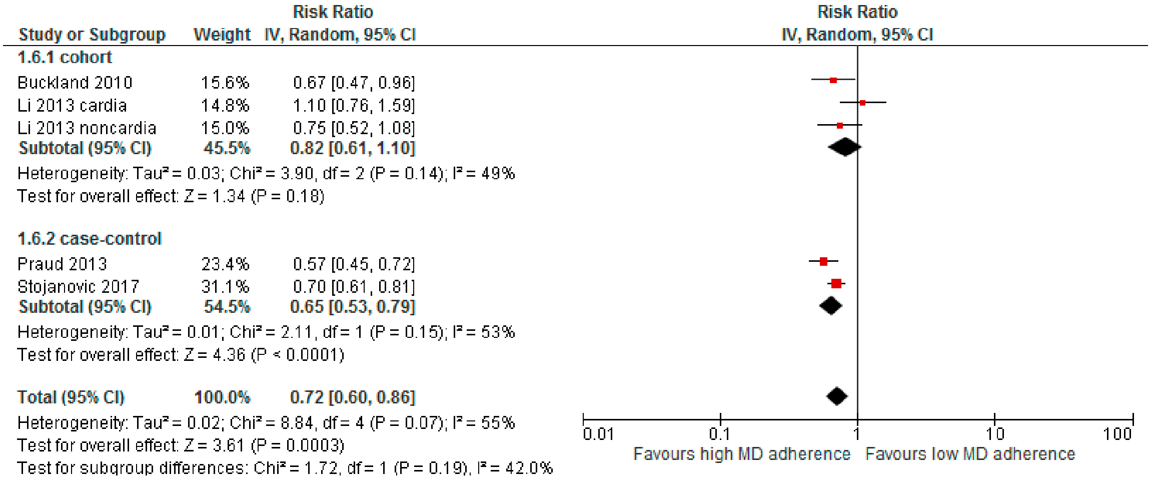


Supplementary Figure S7. Forest plot showing pooled risk ratio (RRs) with 95% CI for risk of gastric cancer for two cohort studies and two case-control study.

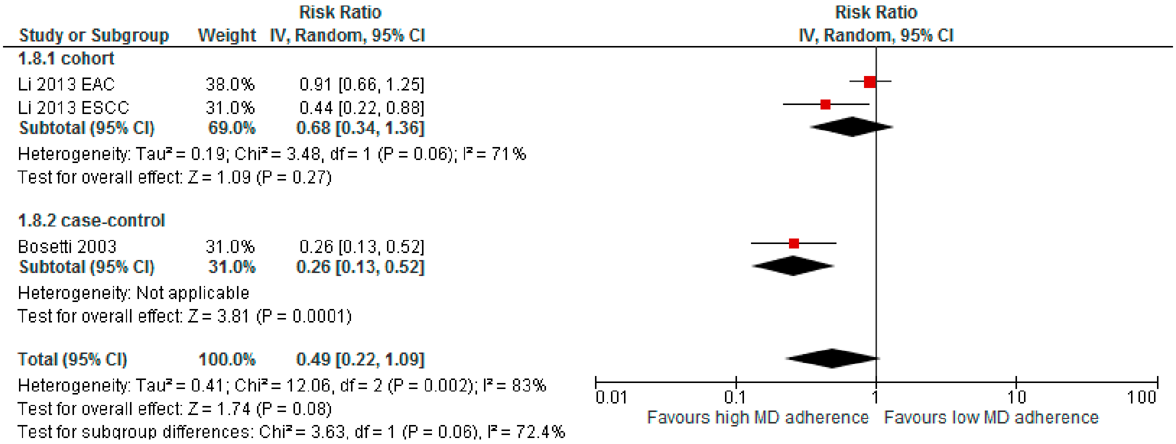


Supplementary Figure S8. Forest plot showing pooled risk ratio (RRs) with 95% CI for risk of esophageal cancer for one cohort study and one case-control study.

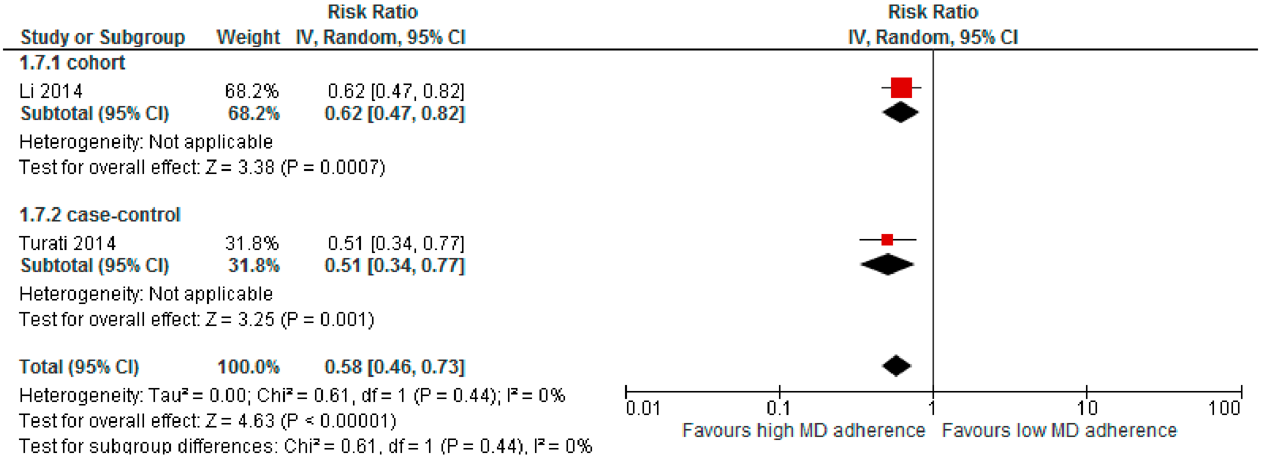


Supplementary Figure S9. Forest plot showing pooled risk ratio (RRs) with 95% CI for risk of liver cancer for one cohort study and one case-control study.

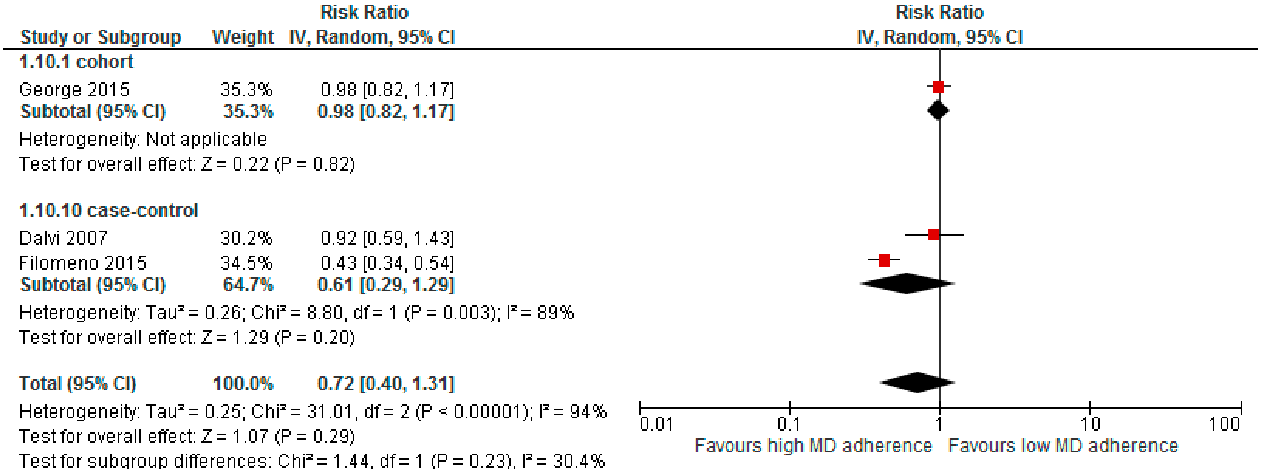


Supplementary Figure S10. Forest plot showing pooled risk ratio (RRs) with 95% CI for risk of endometrial cancer for one cohort study, and two case-control studies.

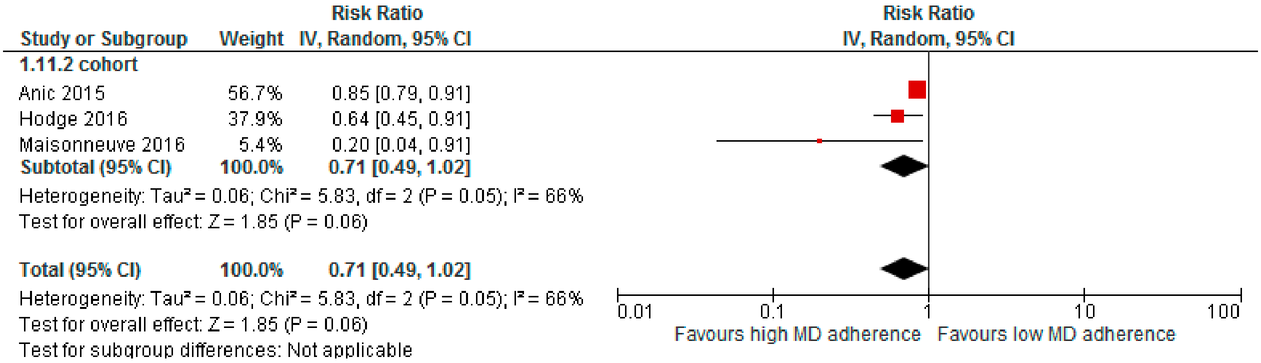


Supplementary Figure S11. Forest plot showing pooled risk ratio (RRs) with 95% CI for risk of respiratory cancer for three cohort studies.

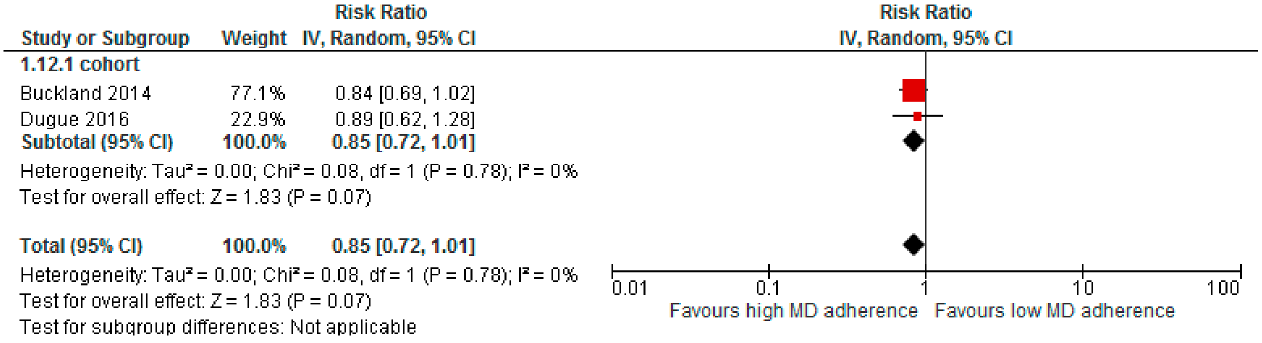


Supplementary Figure S12. Forest plot showing pooled risk ratio (RRs) with 95% CI for risk of bladder cancer for two cohort studies.

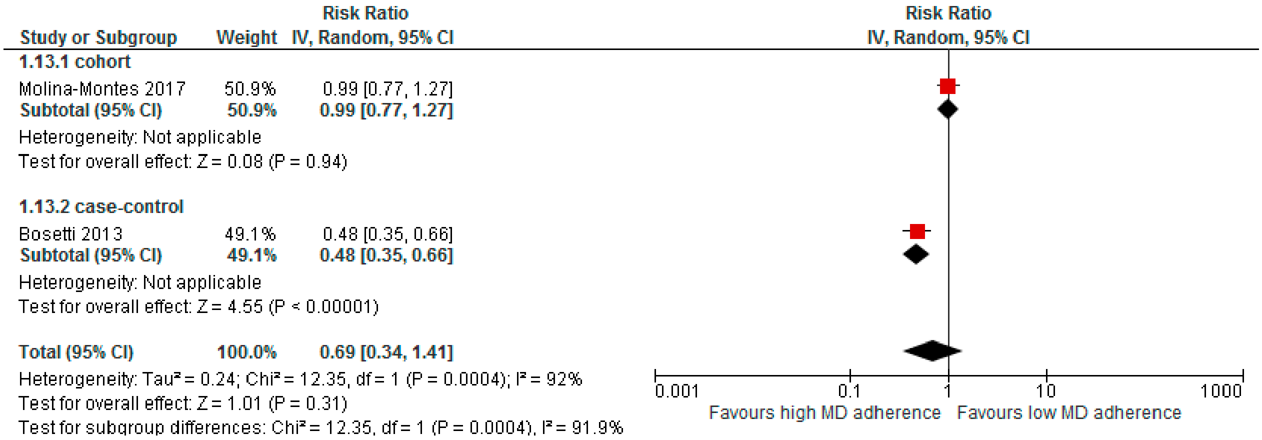


Supplementary Figure S13. Forest plot showing pooled risk ratio (RRs) with 95% CI for risk of pancreatic cancer for one cohort study and one case-control study.

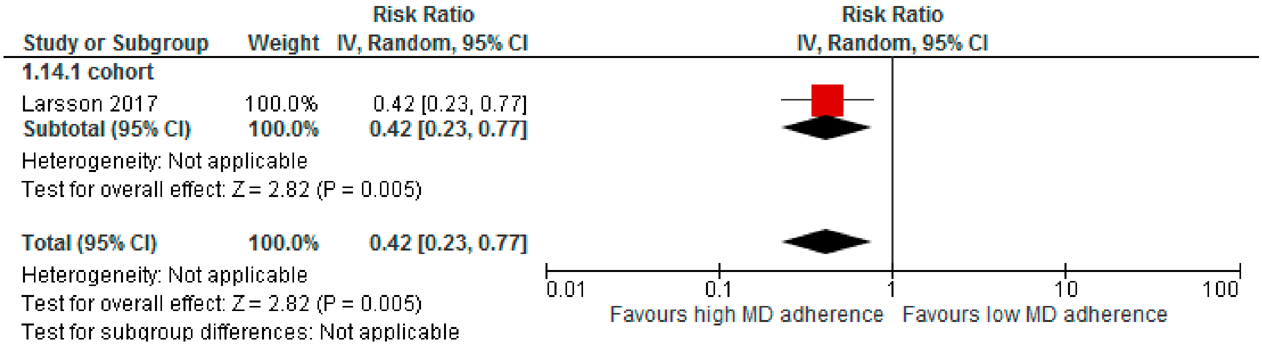


Supplementary Figure S14. Forest plot showing pooled risk ratio (RRs) with 95% CI for risk of gallbladder cancer for one cohort study.

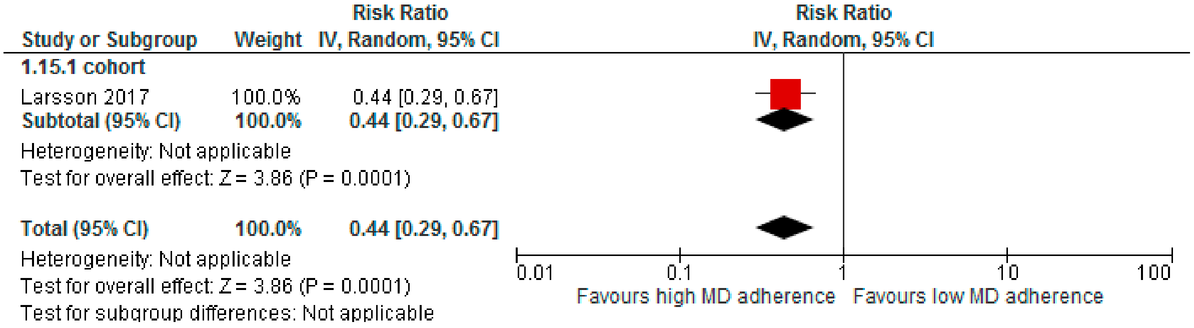


Supplementary Figure S15. Forest plot showing pooled risk ratio (RRs) with 95% CI for risk of biliary tract cancer for one cohort study.

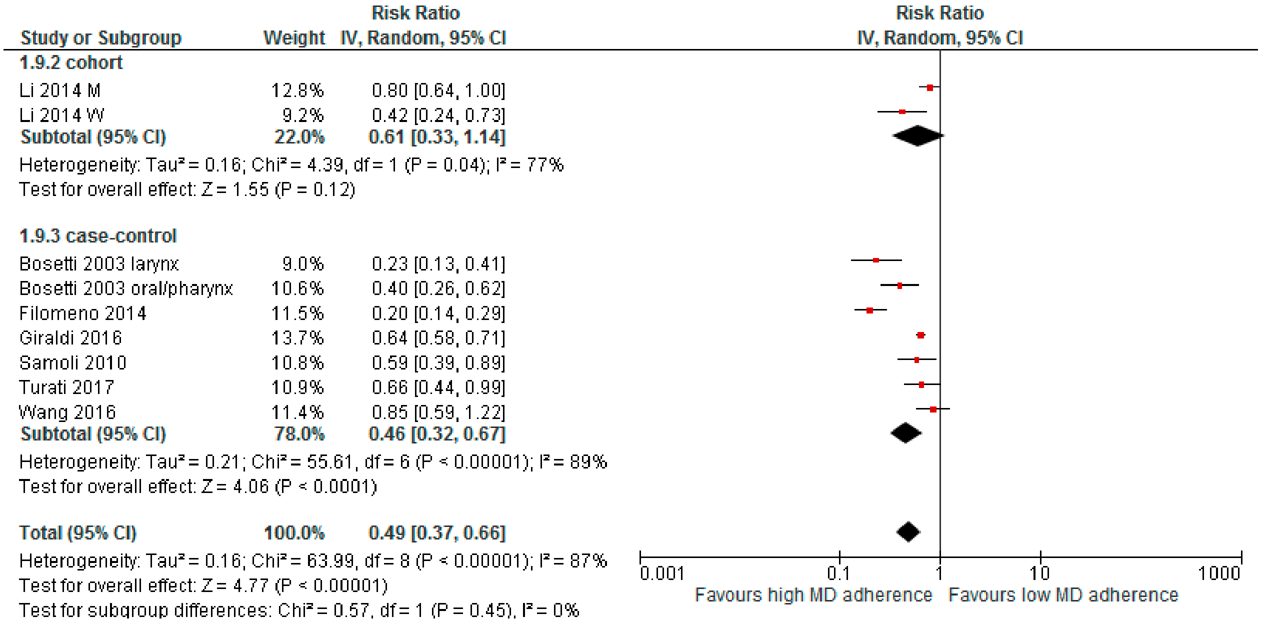


Supplementary Figure S16. Forest plot showing pooled risk ratio (RRs) with 95% CI for risk of head and neck cancer for one cohort study and six case-control studies.

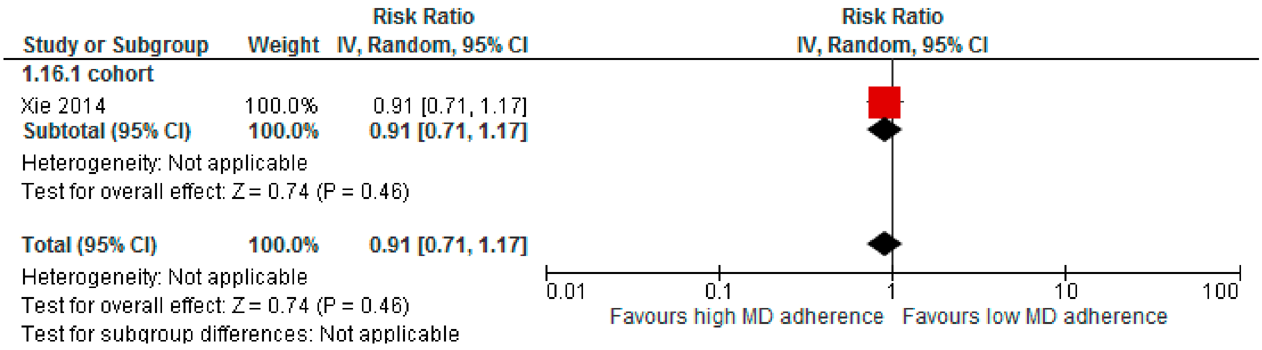


Supplementary Figure S17. Forest plot showing pooled risk ratio (RRs) with 95% CI for risk of ovarian cancer for one cohort study.

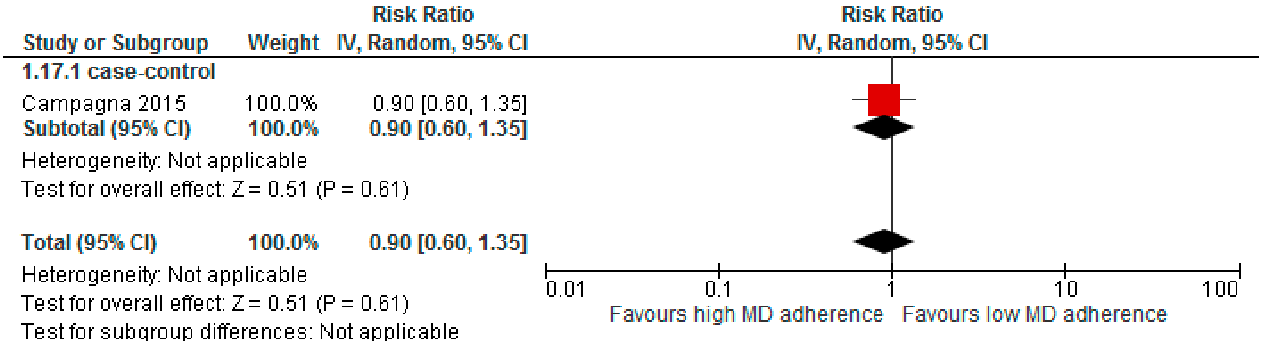


Supplementary Figure S18. Forest plot showing pooled risk ratio (RRs) with 95% CI for risk of lymphoma for one case-control study.

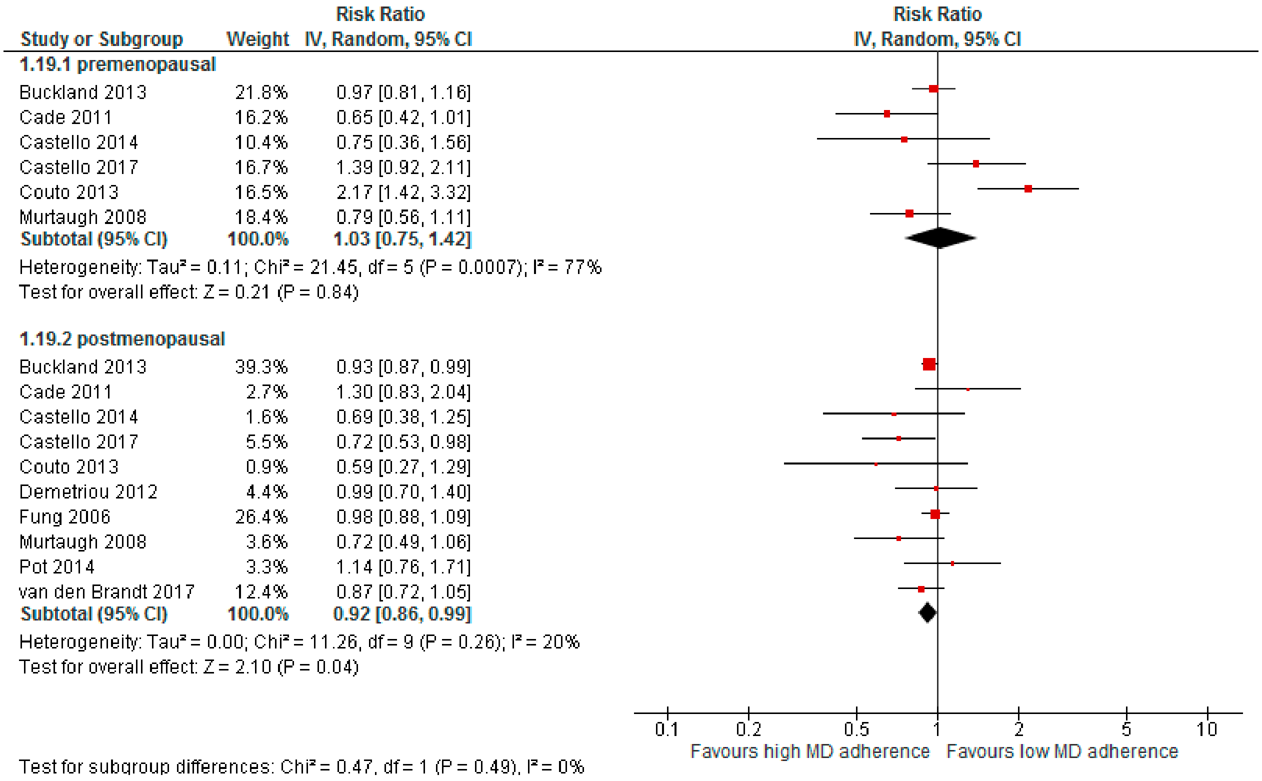


Supplementary Figure S19. Forest plot showing pooled risk ratio (RRs) with 95% CI for risk of breast cancer for pre vs. postmenopausal women.

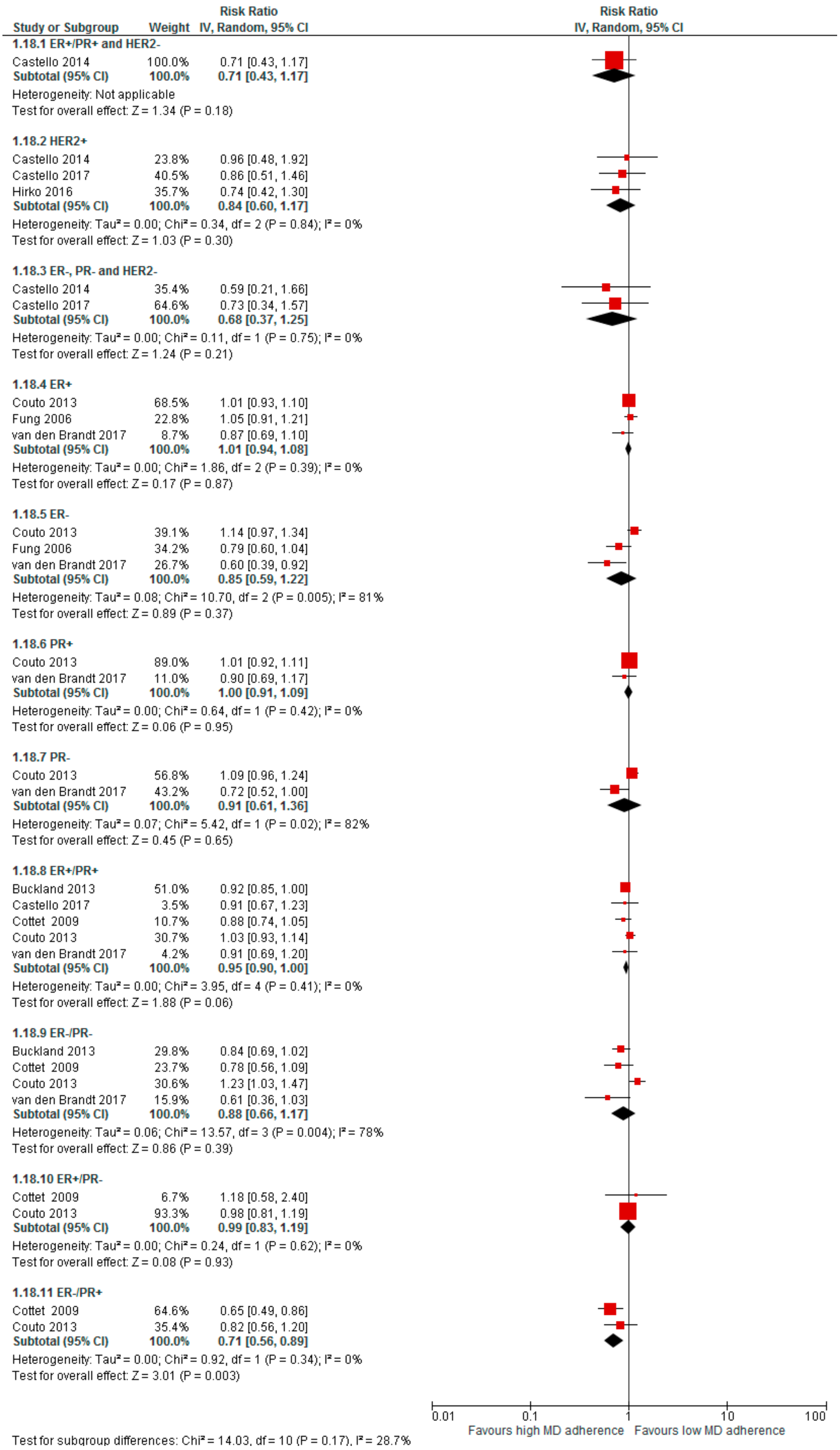


Supplementary Figure S20. Forest plot showing pooled risk ratio (RRs) with 95% CI for risk of breast cancer for breast cancer types.

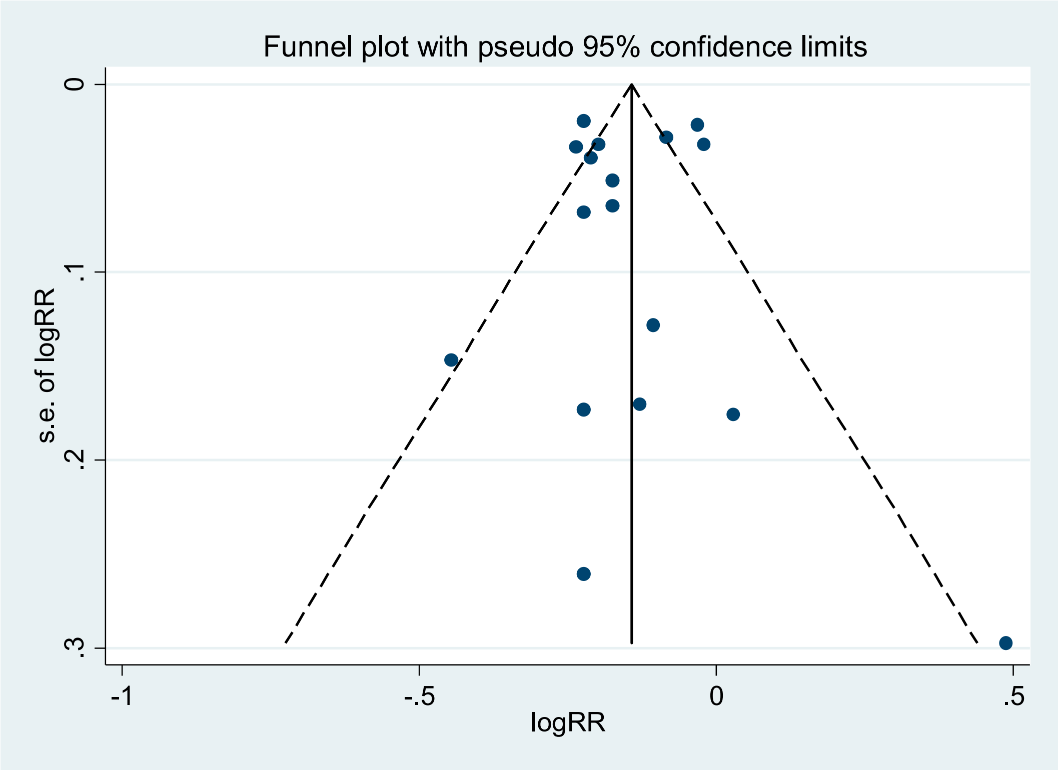


Supplementary Figure S21: Funnel plot showing study precision against the relative risk effect estimate with 95% CIs for cancer mortality. SE = Standard error

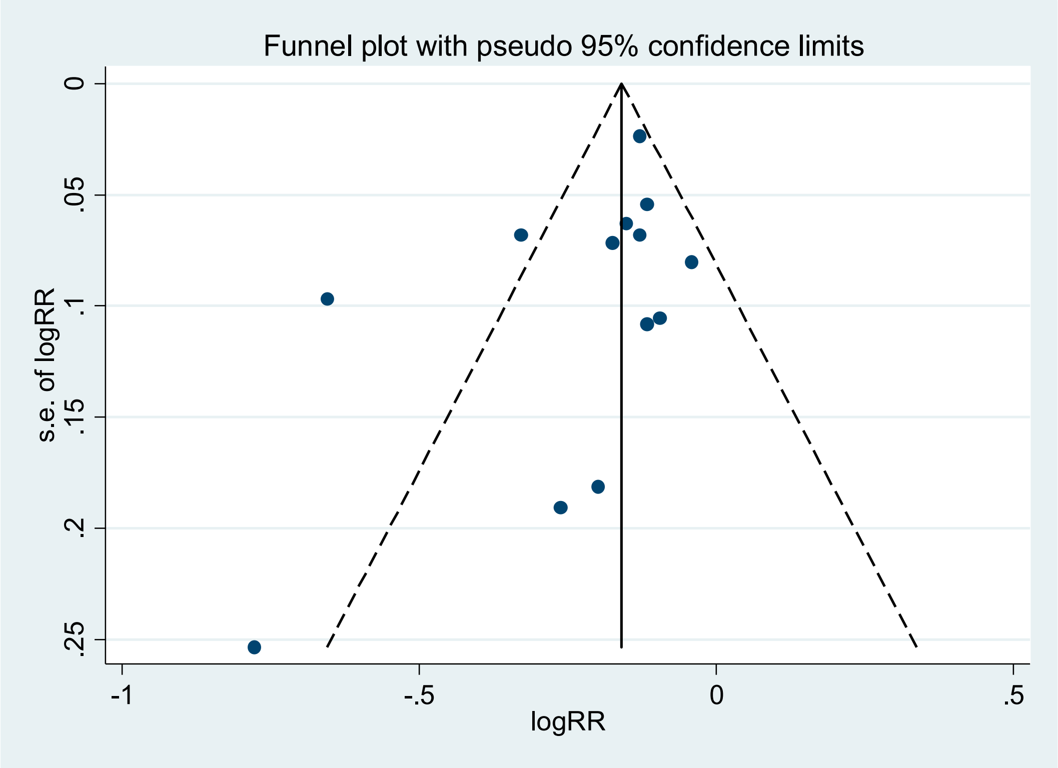


Supplementary Figure S22: Funnel plot showing study precision against the relative risk effect estimate with 95% CIs for colorectal cancer. SE = Standard error

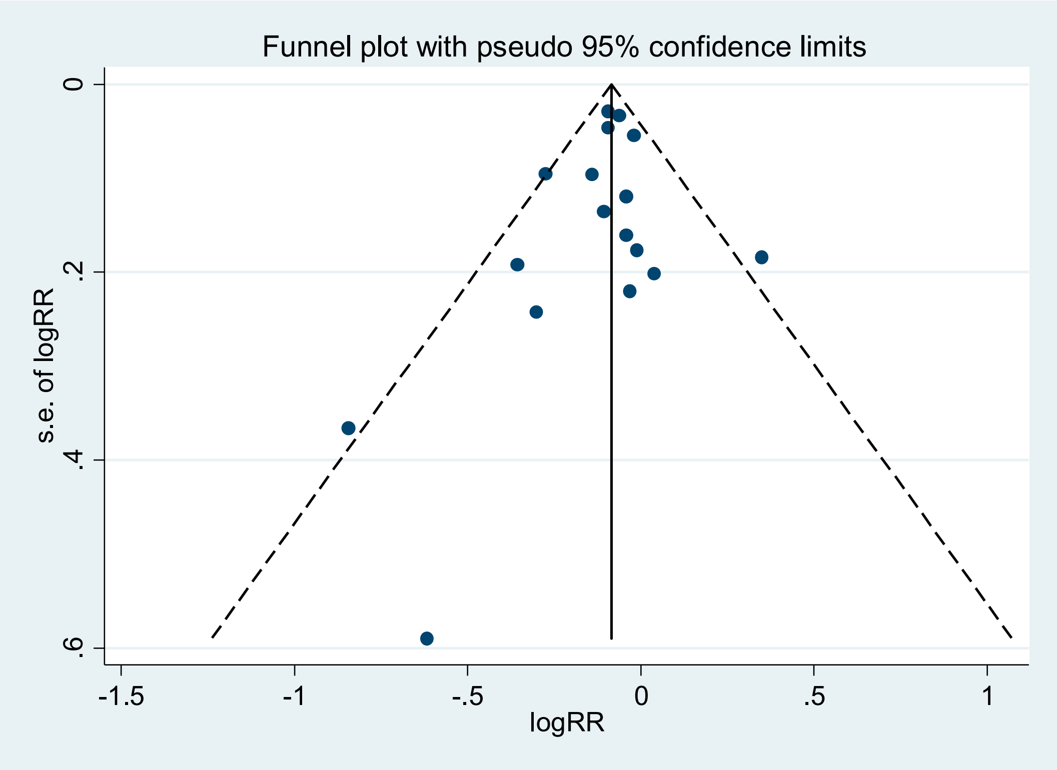


Supplementary Figure S23: Funnel plot showing study precision against the relative risk effect estimate with 95% CIs for breast cancer. SE = Standard error
